# Supplementary material for: Induction of labour for predicted macrosomia: study protocol for the ‘Big Baby’ randomised controlled trial
Source: BMJ Open. 2022 Nov 11;12(11):e058176. doi: 10.1136/bmjopen-2021-058176 (PMC9660609; doi:10.1136/bmjopen-2021-058176)
Supplement: Supplementary data [file bmjopen-2021-058176supp001.pdf]

[Print on Trust Headed Paper]

Participant Information Sheet for the Big Baby Trial

This information sheet is available in large print, audio and minority language translations. For copies, please email: BigBaby@warwick.ac.uk or download them from the website: http://warwick.ac.uk/bigbaby.

Trial title

Induction of labour for predicted macrosomia -The 'Big Baby Trial'.

'Macrosomia' refers to babies who appear to be bigger than expected.

Invitation and brief summary

Your recent ultrasound scan shows that your baby appears bigger than expected. We are inviting you to take part in a research trial to find out the best time to deliver bigger babies. We are aiming for 4000 women across the UK to take part in the research trial.

Before you decide if you want to take part in the trial, please read this information sheet carefully – it explains why the research is being done and what it means for you if you take part. One of our team will go through the information sheet with you and answer any questions you have. You can also discuss the research trial with the obstetrician or midwife looking after you.

What is the trial about?

The purpose of this trial is to find out if 'inducing' (starting) labour earlier than usual, at 38 weeks, makes it less likely that 'shoulder dystocia' will happen in women whose babies appear to be bigger than expected (over the 90th centile on the growth chart).

'Shoulder dystocia' is when the baby's head has been born but one of the shoulders becomes stuck behind the woman's pubic bone (one of the bones in the pelvis), delaying the birth of the baby's body.

'Over the 90th centile' Your growth chart is created to estimate the ideal weight your baby should be for your size and ethnicity. One in 10 babies will be bigger than expected (referred to as 'over the 90th centile').

Women who are told they may have a big baby following their antenatal ultrasound scan will not necessarily have a big baby by the time their baby is delivered.

In most cases, women with big babies have a normal labour and birth and there are no concerns. However, there is an increased chance that the birth may be more difficult, which could result in shoulder dystocia. Shoulder dystocia happens in one in 150 of all vaginal births. We know that shoulder dystocia happens more often in bigger babies, but we cannot be certain how often. We estimate that for big babies, shoulder dystocia could happen in up to one in 25 vaginal births. If shoulder dystocia happens, the midwives and doctors will use different ways to help to free the baby's shoulders, which usually allows the body to be born.

Ewington LJ, *et al. BMJ Open* 2022; 12:e058176. doi: 10.1136/bmjopen-2021-058176

[Print on Trust Headed Paper]

Most babies who experience shoulder dystocia will be fine with no complications. But in around one in 10 cases of shoulder dystocia, there is stretching of the nerves in the baby's neck (brachial plexus injury). This can cause loss of movement in the baby's arm. In most cases this loss of movement is temporary, but in one in 10 of those babies the loss of movement can be permanent. In some cases, the baby may have a broken collarbone, but this heals quickly and easily in babies.

We currently do not know the best way to deliver bigger babies. It may be that starting labour earlier, when babies are smaller, means that shoulder dystocia is less likely to happen. This issue has been identified as an important unanswered question for NHS maternity units. The results of this trial will help women, midwives and obstetricians decide on the best way to deliver big babies.

What will happen if I agree to take part?

You will meet with a member of our research team at the maternity unit, either face-to-face or by telephone or video consultation, who will explain the trial. You are welcome to involve your partner, family member or friend in this discussion. We will ask you about your medical history, any previous pregnancies, and your current pregnancy. We will also review your ultrasound scan results. You can ask our team member any questions you may have. If you are eligible and would like to take part, we will ask you to either sign a consent form or to give your consent during a telephone or video call (we will then fill in the consent form for you following your verbal consent and give you a copy of this).

After you have agreed to take part in the trial and provided your consent, we will ask you to fill in questionnaires about your health, well-being and quality of life. You will be randomly selected to either have your labour induced at around 38 weeks (**the intervention group**) or to continue as normal (**the standard care group**).

If you are randomly selected to have your labour induced, your midwife or obstetrician will organise a time and date for your labour to be started (induction) and they will explain how and when this will happen.

If you are in the standard care group, you will receive the usual standard care provided by your hospital and will attend your usual antenatal appointments.

If at any point your obstetrician or midwife feels that a different plan needs to be made for your birth they will discuss this with you. If you no longer feel happy about the birth options you have as part of the trial and would like to discuss other options, you can discuss these with your obstetrician or midwife. If your birth is different to the one you were allocated, for example you have a caesarean section, you can still continue to be part of the trial.

If you agree to take part, we will collect information from your hospital records about the birth of your baby and about your and your baby's health during the time you are in hospital. We will contact you two and six months after your baby is born and ask you to fill in questionnaires about your and your baby's health and well-being. Please see page 6 of this information sheet for more details about the information we will collect.

Ewington LJ, *et al. BMJ Open* 2022; 12:e058176. doi: 10.1136/bmjopen-2021-058176

[**Print on Trust Headed Paper**]

What are the clinical alternatives?
All women in the trial will receive the same care that they would have received if the trial was not happening. Even if you do not want to join the full trial, you can still take part in the research. (This is known as the 'cohort study'.) If you agree to join the cohort study, we would like to collect information about your baby's birth to help doctors and midwives make decisions about the best way to deliver big babies in the future. If you are happy for us to collect information about your baby's birth, we will ask you to sign a consent form.

Some women with big babies may decide that they would like their labour to be induced or that they would like to wait for it to start naturally. If you are sure that you want your labour to be induced or that you would like to wait for it to start naturally, please discuss this with your midwife and obstetrician.

If you would like to have your baby by caesarean section, you will have an opportunity to discuss this with your midwife and obstetrician. If you have a caesarean section, we would like to find out information about your birth and also about your and your baby's health after the birth. If you are happy for us to collect this information, we will ask you to sign a consent form. We will also ask you to fill in questionnaires that will include questions about your and your baby's health, well-being and quality of life. We will ask you to fill in a questionnaire when you first agree to take part in the trial and again two and six months after your baby is born. If you tell us your baby has had important health problems over this time, we will collect information about these problems from their hospital and GP records. This will help us to better understand and compare the risks and benefits of a vaginal birth or caesarean section in women with bigger babies.

What are the possible benefits of taking part in the trial?
We do not know if taking part in the research trial would benefit you or your baby. The findings will help us to advise women in the future on the best way to deliver their babies to reduce possible problems during the birth, including the risk of shoulder dystocia.

What are the possible disadvantages and risks of taking part?
Giving birth in the UK is generally very safe, whichever type of birth you have. However, if your baby is big, there can be increased risks to both you and your baby. In this research we are trying to find out the best way to reduce these risks in women who have a normal (vaginal) birth. Sometimes, obstetricians recommend a caesarean section instead of a vaginal birth, and some women may choose to have a caesarean section.

If you have a caesarean section there are different risks to consider. We have summarised what we know about the risks of vaginal births, inducing labour and caesarean sections in the tables below, but at the moment we do not know which type of birth has the fewest risks for women with bigger babies. Your midwife or obstetrician will be able to discuss the risks with you in more detail.

Ewington LJ, *et al. BMJ Open* 2022; 12:e058176. doi: 10.1136/bmjopen-2021-058176

BigBaby CLINICAL TRIAL

[Print on Trust Headed Paper]

Table 1 Risks of a vaginal birth with a big baby

Risks to the baby	Risks to the woman
We do not know for certain how many big babies will experience shoulder dystocia. We estimate that up to one in 25 big babies will experience shoulder dystocia and will need extra help to deliver their shoulders. Most babies who experience shoulder dystocia will have no long-term effects.	Sometimes the labour can be longer for bigger babies. In the UK, 15 in 100 women who are planning to have a vaginal birth will need to have an emergency caesarean section (please see table 3 below). Some women may need to have a forceps or ventouse (suction) delivery.
One in 10 babies who experience shoulder dystocia will have stretching of the nerves in the neck. This is called brachial plexus injury and can causes loss of movement in the baby's arm. The most common type of brachial plexus injury is Erb's palsy. For one in 10 babies with a brachial plexus injury, the loss of movement will be permanent.	Three in 100 women will have a tear to their vagina that extends into the back passage. This could affect their bowel control if the tear is not identified and repaired.
In babies who experience shoulder dystocia, one in 10 may have a fracture to their collarbone. Four in 100 babies who experience shoulder dystocia may have a fracture to their arm. These heal well.	Sometimes women with a big baby may experience heavier bleeding after the baby is born. In rare cases, some women may need a blood transfusion.
Very rarely, a baby may suffer brain damage if they did not get enough oxygen during the birth because of shoulder dystocia.	

Table 2 Risks of inducing labour with a big baby

Risks to the baby	Risks to the woman
Inducing labour at 38 weeks is safe for the baby. There is some evidence that inducing labour earlier can lead to jaundice in the baby. This usually has no long-term effects.	Often women who have labour induced will find their labour is longer and more painful than for women who go into labour naturally.
This trial aims to find out if inducing labour early, at 38 weeks, reduces the chance of shoulder dystocia. If the baby experiences shoulder dystocia, the possible complications are shown in table 1.	If you have a vaginal birth the risks are shown in table 1. Having labour induced can increase the risk of a tear to your vagina that extends into your back passage.
Babies who are born one or two weeks early are slightly more likely to need extra help at school, for example help with reading. This would affect less than 1% of babies born at 38 weeks compared with those born at 40 weeks.	Sometimes if you are being induced you may need an emergency caesarean section, and the risks of this are shown in table 3.

Ewington LJ, *et al. BMJ Open* 2022; 12:e058176. doi: 10.1136/bmjopen-2021-058176

[Print on Trust Headed Paper]

Table 3 Risks of caesarean section

Risks to the baby	Risks to the woman
One in 10 babies may experience breathing difficulties. Some of these babies will need to have treatment for this in the neonatal unit.	Nine in 100 women report persistent pain at the wound site and in their abdomen for a few months following a caesarean section.
One to two babies in 100 will have a cut to their skin.	Five in 100 women will need to be readmitted to hospital following a caesarean section. This might be because their wound isn't healing or because they have an infection.
Some women report that it takes longer to bond with their baby after a caesarean section.	Six in 100 women will have an infection after a caesarean section. The infection may involve the scar, their bladder or kidneys, or the lining of their womb.
	One in 1000 women may have an injury to their bladder or bowel during a caesarean section. This will need repairing.
	Five in 1000 women bleed heavily (haemorrhage) during a caesarean section. Some of these women will need to have a blood transfusion. In some cases, a woman may need to have a hysterectomy (where the womb is removed) to control the bleeding.
	Five in 1000 women may need to have further surgery after their caesarean section.
	Six in 10,000 women will have a blood clot in their leg or lung following a caesarean section.
	One in four women who have a caesarean section will need another caesarean section if they attempt a vaginal birth in their next pregnancy. If you have a caesarean section and decide to try a vaginal birth in your next pregnancy, you would need extra monitoring in labour as there is a risk (one in 200 women) that the scar in the uterus can open during labour.
	If you have a caesarean section in this pregnancy, in your next pregnancy there is an increased chance of a stillbirth. This is uncommon.
	If you have a caesarean section in this pregnancy and the placenta is low in your next pregnancy, there is an increased chance that the placenta will not come away easily after the baby has been born. This can cause serious bleeding and may mean you need to have a hysterectomy. This is uncommon, but the chance increases with each caesarean section.

Ewington LJ, *et al. BMJ Open* 2022; 12:e058176. doi: 10.1136/bmjopen-2021-058176

[Print on Trust Headed Paper]

What other information will you collect?
University Hospitals Coventry and Warwickshire (UHCW) are the sponsor for this trial. The trial will be managed by Warwick Clinical Trials Unit at the University of Warwick (UoW). UHCW and UoW will use information you provide and information from your hospital records and your GP records to carry out this trial. UHCW will act as the data controller for this trial, which means that they are responsible for looking after the information we collect about you and for making sure we are using it properly. UoW will act as a data processor and be under the instruction of UHCW. The trial sites are also data processors and will also be under the instruction of UHCW. You can find out more about how your information is used, how to exercise your rights relating to processing personal information, and the contact details of the data protection officer at https://www.uhcw.nhs.uk/privacy/.

We will collect information from your hospital records about the birth of your baby and about your and your baby's health during the time you are in hospital. This will include ethnic origin and health information, which is regarded as 'special category personal data'. To protect your rights, we will use the minimum amount of personally identifiable information possible. We will collect your name, date of birth, address, phone number and email address from your medical records, so we can contact you about the research and make sure that relevant information about the trial is recorded for your care, and to oversee the quality of the research. In order to do this, individuals from UHCW, UoW and regulatory authorities may review your medical notes and research records.

We will also use your information to contact you two and six months after your baby is born to ask you to fill in questionnaires about your and your baby's health and well-being, and about what, if any, healthcare services you and your baby have used. We may contact you by post, telephone, email or text message. If you tell us that your baby has had important health problems over this time, with your permission, we will collect information about these from your or your baby's hospital and GP records, or we may contact you to discuss these problems further. When we receive your questionnaire, we will review your information and if we find that any important details are missing we will contact you to collect this.

If you have given permission, we may also contact you and ask if you are willing to take part in a telephone or face-to-face interview with a member of our research team about your experiences of taking part in the Big Baby Trial. With your permission, we would also like to interview your partner or birth partner to understand what their experience of taking part in the Big Baby Trial was like.

In the UK, it is very rare that a woman dies during late pregnancy or during or after the birth (this risk is less than one in 10,000). It is also uncommon that a baby dies in the first 28 days after the birth (the risk is less than one in 400). If either of these things happens while you are taking part in this trial, it is important to us that we try to keep any distress to you and your family to a minimum. To help us achieve this, with your permission, we will check your baby's hospital records to check that your baby is alive before we invite you to fill in the two questionnaires (two and six months after the birth) or invite you to take part in an interview or further studies. When we check your baby's records, if we find that your baby has died

Ewington LJ, et al. BMJ Open 2022; 12:e058176. doi: 10.1136/bmjopen-2021-058176

we will not contact you. It is important for us to know if you or your baby has died and if so, what caused this. To give us access to this information, we would like your permission to look at linked information held by an organisation called NHS Digital, who look after healthcare information. We would only collect this information if you did not fill in your two and six month follow-up questionnaires. We will collect your and your baby's NHS numbers from your medical records to do this.

If you have not filled in the two month follow-up questionnaire by the time that we send out the six month questionnaire, we will invite you to fill in a combined two- and six- month follow-up questionnaire six months after the birth.

If you have given us permission, we may also contact you when your child reaches age 16 to ask for their permission to keep their contact details. We would like to keep their contact details in case we want to do more research in the future.

Do I have to take part?

It is entirely up to you whether or not you take part in the trial or any other part of the research. You do not have to take part and there will be no difference in any aspects of the care that you receive if you choose not to take part. If you want to take part, you will have an opportunity to discuss this sheet with us, and ask us any questions you may have. We will then ask you to sign a consent form to confirm you have agreed to take part. Even after agreeing to take part, if you change your mind you can withdraw from the trial at any time, without having to give a reason. This will not affect the care you receive.

What happens when the research trial stops?

At the end of the trial, which will take 60 months, we will analyse the information we have collected to decide if starting labour early is the best thing to do for women and their babies. In the future, these results will help women who are expecting big babies decide if they should be induced.

Whichever part of the trial you join, we would like to keep the information we hold on you and your baby after the end of the trial. This is so that we can contact you as your baby is growing up (or we can contact your child when he or she reaches age 16) to find out if anything related to the birth has affected their longer-term health. So that we can do this, we would like your permission to look at Hospital Episode Statistics for you or your child (or both). We will collect your and your baby's NHS number to do this.

Expenses and payments

We expect that research visits will be in-line with your routine clinic appointments, so you will not need to make any extra trips to hospital. There will be no payments or travel expenses for taking part in this research.

What if I have a concern?

If you have any concerns, please talk to a member of the research team (details below) or your obstetrician or midwife. They will provide you with all the information you ask for. If you are still not happy, you should contact the<< insert as appropriate>>, who can offer

Ewington LJ, *et al. BMJ Open* 2022; 12:e058176. doi: 10.1136/bmjopen-2021-058176

confidential guidance on how to get independent advice. You will have the same legal rights as any other person treated in the NHS. If you or your baby is harmed by negligence you may have grounds for legal action, but you may have to pay any costs involved.

What will happen if I don't want to carry on with the trial?

Taking part in the research trial is entirely voluntary. If you do not want to continue in the trial, you can withdraw at any time without giving a reason and without it affecting your care in any way. If you decide to withdraw from the trial you can choose to have no further contact from us. However, we will keep the information about you that we have already collected if you do this. Your rights to access, change or move your information are limited as we need to manage your information in specific ways in order for the research to be reliable and accurate.

Will information about me and my baby be kept confidential?

Yes. All information we collect about you and your baby is strictly confidential. Once you have agreed to take part in the trial, we will store your contact details in a secure database which the trial team can access. Research information we collect for the trial will refer to you by a unique trial number, so the risk of you being identified is very low. We will hold contact details and research information in separate parts of the database. In rare circumstances a senior researcher from the trial team may need to pass on information we receive during the trial if there is a concern about a significant risk of harm to you or your baby, or to other people. They will only pass information to a person with authority to deal with such concerns and if possible the researcher will explain to you what information they are passing on and why. All information will be stored securely and held at the Perinatal Institute and the Warwick Clinical Trials Unit, in line with all relevant UK laws, and only authorised staff will have access to it.

When you agree to take part in a research trial, we may give information about your health and care to researchers who are running other research studies in this organisation and in other organisations. These other organisations may be universities, NHS organisations or companies involved in health and care research in this country or abroad. Organisations and researchers will only use your information to carry out research in line with the UK Policy Framework for Health and Social Care Research. The information we share will not identify you and will not be combined with other information in a way that could identify you. The information will only be used for the purpose of health and care research. It will not affect your care, and organisations and researchers cannot use it to contact you. Your information will not be used to make decisions about future services that are available to you, such as insurance.

Your rights to see, change or move your information are limited, as your information is managed in specific ways to make sure the research is reliable and accurate. If you withdraw from the trial, we will keep any information we have already collected about you. To protect your rights, we will collect as few details as possible that could identify you.

To find out more about how your information is handled, you can visit the privacy notices of the data controllers (those responsible for how and why your personal information is collected, used and held).

Ewington LJ, *et al. BMJ Open* 2022; 12:e058176. doi: 10.1136/bmjopen-2021-058176

[Print on Trust Headed Paper]

www.uhcw.nhs.uk/privacy/
www.warwick.ac.uk/services/idc/dataprotection/privacynotices/researchprivacynotice

Who will be able to see my information?
Occasionally we will access your or your baby's medical records to make sure the information we have collected about you both is accurate. Only authorised staff will do this. The people who analyse the information will not be able to identify you. We will ask for your permission to tell your GP that you are taking part in the clinical trial. If you do not want us to tell your GP, you will not be able to take part.

Only authorised staff will have access to your personal details and be able to trace your identity. At the end of the study, we will store the information we collect for the trial about you and your baby for at least 25 years if you are in the randomised trial or at least 10 years for the cohort study. This is in line with UK law.

What will happen to the results of this trial?
Once the trial is complete, we will prepare and publish a report. The results will be available to the hospitals that took part in the trial. We may share information relating to the trial in scientific meetings and it may be published in scientific journals. You will not be identified in any reports or publications and none of the information will be able to be traced to you personally. The results of the trial will be published on the Big Baby website http://warwick.ac.uk/bigbaby .

Who is organising and funding this trial?
The trial is funded by the National Institute for Health Research (NIHR), Health Technology Assessment Programme. The Government set up the NIHR in 2006 to provide organised funding for research within the NHS. University Hospital Coventry and Warwickshire NHS Trust is sponsoring the trial. This covers the insurance and indemnity costs that apply to research trials. Professor Siobhan Quenby (from University Hospital Coventry & Warwickshire NHS Trust and The University of Warwick) and Professor Jason Gardosi (from the Perinatal Institute) are the chief investigators and have overall responsibility for the trial. The University of Warwick Clinical Trials Unit is organising the administration of the trial.

Who has reviewed this trial?
The trial was reviewed and approved by South West - Exeter Research Ethics Committee (REC) on 1st February 2018. The REC are an independent group of people who review all research carried out in the NHS to protect your safety, rights, well-being and dignity.

What if I want to complain?
If you want to make a complaint, please contact: Research and Development, 4th Floor Rotunda, ADA40007, University Hospitals Coventry and Warwickshire NHS Trust, Clifford Bridge Road, Coventry, CV2 2DX.

Ewington LJ, *et al. BMJ Open* 2022; 12:e058176. doi: 10.1136/bmjopen-2021-058176

[Print on Trust Headed Paper]

How can I contact the hospital research team?
The hospital research team will be happy to answer any questions about the trial or your involvement in it, either now or in the future, please contact the hospital research team.
Email: <<Please insert>>
Phone: <<Please insert>>
Write to: <<Please insert>>

For more information about the 'Big Baby' trial and other useful information, please visit the Big Baby Project website: http://warwick.ac.uk/bigbaby.

Thank you for taking time to read this information sheet.

Funding acknowledgment and disclaimer - This project is funded by the National Institute for Health Research Health Technology Programme 16/77/02. The views expressed are those of the author and not necessarily those of the NIHR or the Department of Health and Social Care.
