# Supplementary material for: Induction of labour for predicted macrosomia: study protocol for the ‘Big Baby’ randomised controlled trial
Source: BMJ Open. 2022 Nov 11;12(11):e058176. doi: 10.1136/bmjopen-2021-058176 (PMC9660609; doi:10.1136/bmjopen-2021-058176)
Supplement: Author's manuscript [file bmjopen-2021-058176.draft_revisions.pdf]

BMJ Open

BMJ Open is committed to open peer review. As part of this commitment we make the peer review history of every article we publish publicly available.

When an article is published we post the peer reviewers' comments and the authors' responses online. We also post the versions of the paper that were used during peer review. These are the versions that the peer review comments apply to.

The versions of the paper that follow are the versions that were submitted during the peer review process. They are not the versions of record or the final published versions. They should not be cited or distributed as the published version of this manuscript.

BMJ Open is an open access journal and the full, final, typeset and author-corrected version of record of the manuscript is available on our site with no access controls, subscription charges or pay-per-view fees (http://bmjopen.bmj.com).

If you have any questions on BMJ Open's open peer review process please email info.bmjopen@bmj.com

BMJ Open

Induction of Labour for Predicted Macrosomia: Study Protocol for the 'Big Baby' Randomised Controlled Trial

Journal:	*BMJ Open*
Manuscript ID	bmjopen-2021-058176
Article Type:	Protocol
Date Submitted by the Author:	08-Oct-2021
Complete List of Authors:	Ewington, Lauren; University of Warwick Faculty of Medicine, Biomedical Sciences; University Hospitals Coventry and Warwickshire NHS Trust, Women's and Children's<br>Gardosi, Jason; Perinatal Institute<br>Lall, Ranjit; University of Warwick, Warwick Clinical Trials Unit<br>Underwood, Martin; Warwick University, Warwick Clinical Trials Unit; University Hospitals Coventry and Warwickshire NHS Trust<br>Fisher, Joanne; University of Warwick Warwick Medical School, Clinical Trials Unit<br>Wood, Sara; University of Warwick, Warwick Clinical Trials Unit<br>Griffin, Ryan; University of Warwick, Warwick Clinical Trials Unit<br>Harris, Kirsten; University of Warwick<br>Bick, Debra; University of Warwick, Warwick Clinical Trials Unit, Warwick Medical School<br>Booth, Katie; University of Warwick, Warwick Clinical Trials Unit<br>Brown, Jaclyn; University of Warwick, Clinical Trials Unit<br>Butler, Emily; Perinatal Institute<br>Fowler, Kelly; Perinatal Institute<br>Williams, Mandy; Perinatal Institute<br>Deshpande, Sanjeev; Shrewsbury and Telford Hospital NHS Trust<br>Gornall, Adam; Shrewsbury and Telford Hospital NHS Trust<br>Dewdney, Jackie; Erb's Palsy Group<br>Hillyer, Karen; Erb's Palsy Group<br>Gates, Simon; University of Birmingham, Cancer Research UK Clinical Trials Unit<br>Jones, Ceri; University Hospitals Coventry and Warwickshire NHS Trust<br>Mistry, Hema; University of Warwick, Warwick Clinical Trials Unit<br>Petrou, Stavros; Nuffield Department of Primary Care Health Sciences<br>Slowther, Anne-Marie; University of Warwick, Social Sciences and Systems in Health<br>Willis, Adrian; University of Warwick, Warwick Clinical Trials Unit<br>Quenby, Siobhan; University of Warwick; University Hospitals Coventry and Warwickshire NHS Trust
Keywords:	OBSTETRICS, MEDICAL ETHICS, Ultrasonography < OBSTETRICS

For peer review only - http://bmjopen.bmj.com/site/about/guidelines.xhtml

Induction of Labour for Predicted Macrosomia:

Study Protocol for the 'Big Baby' Randomised Controlled Trial

Lauren Jade Ewington[1,2], Jason Gardosi[3], Ranjit Lall[4], Martin Underwood[2,4], Joanne Fisher[4], Sara Wood[4], Ryan Griffin[4], Kirsten Harris[4], Debra Bick[4], Katie Booth[4], Jaclyn Brown[4], Emily Butler[3], Kelly Fowler[3], Mandy Williams[3], Sanjeev Deshpande[5], Adam Gornall[5], Jackie Dewdney[6], Karen Hillyer[6], Simon Gates[7], Ceri Jones[2], Hema Mistry[4], Stavros Petrou[8], Anne-Marie Slowther[9], Adrian Willis[4], Siobhan Quenby[1,2]

Author Affiliations:

1. Division of Biomedical Sciences, University of Warwick, Coventry, UK
2. University Hospitals Coventry and Warwickshire NHS Trust, Coventry, UK
3. Perinatal Institute, Birmingham, UK
4. Warwick Clinical Trials Unit, University of Warwick, Coventry, UK
5. Shrewsbury and Telford Hospitals NHS Trust, Telford, UK
6. Erb's Palsy Group, Coventry, UK
7. Cancer Clinical Trials Unit, University of Birmingham, Birmingham, UK
8. Nuffield Department of Primary Care Health Sciences, University of Oxford, Oxford, UK
9. Social Sciences and Systems in Health, University of Warwick, Coventry, UK

Corresponding Author:

Professor Siobhan Quenby: s.quenby@warwick.ac.uk

For peer review only

For peer review only - http://bmjopen.bmj.com/site/about/guidelines.xhtml

ABSTRACT

Introduction

Large-for-gestational age fetuses have an increased risk of shoulder dystocia. This can lead to neonatal fractures, brachial plexus injury, hypoxic ischaemic encephalopathy and death. Early induction of labour in women with a fetus suspected to be macrosomic may mitigate the risk of shoulder dystocia. The Big Baby Trial aims to find if induction of labour at 38^{+0}-38^{+4} weeks' gestation, in pregnancies with suspected large-for-gestational age fetuses, reduces the incidence of shoulder dystocia.

Methods and Analysis

The Big Baby Trial is a multicentre, prospective, individually randomised controlled trial of induction of labour at 38^{+0} to 38^{+4} weeks' gestation versus standard care among women whose fetuses have an estimated fetal weight >90th customised centile according to ultrasound scan at 35^{+0} to 38^{+0} weeks' gestation. There is a parallel cohort study for women who decline randomisation because they opt for induction, expectant management or caesarean section. Up to 4,000 women will be recruited and randomised to induction of labour or to standard care. The primary outcome is the incidence of shoulder dystocia; assessed by an independent expert group, blind to treatment allocation, from delivery records. Secondary outcomes include birth trauma, fractures, haemorrhage, caesarean section rate and length of inpatient stay. The main trial ran seamlessly following an internal pilot study. A qualitative reporting, health economic evaluation and parallel process evaluation are included.

Ethics and Dissemination

The study received a favourable opinion from the South West – Cornwall and Plymouth Health Research Authority on 23/03/2018 (IRAS project ID 229163).

Trial Registration Number

ISRCTN18229892

For peer review only

STRENGHTS AND LIMITATIONS OF THIS STUDY

- This is the largest trial assessing if induction of labour decreases the incidence of shoulder dystocia in women with a suspected large-for-gestational age fetus.
- The main trial ran seamlessly following an internal pilot study. The trial includes qualitative reporting, and health economic and process evaluations.
- Women declining randomisation and opting for an elective caesarean section can consent to participate in a parallel cohort study to collect maternal and neonatal health outcomes.

For peer review only

For peer review only - http://bmjopen.bmj.com/site/about/guidelines.xhtml

INTRODUCTION

Shoulder dystocia occurs when an infant's head has been delivered vaginally and the shoulder becomes stuck behind a woman's pubic bone. This can lead to maternal and fetal complications. Maternal complications include haemorrhage, third- and fourth-degree perineal tears and psychological sequelae. Infant complications include fractures of the clavicle and humerus, brachial plexus injury, hypoxic ischaemic encephalopathy and death(1-3). Shoulder dystocia and its complications are common indications for litigation in obstetrics with settlements dealt with by the UK NHS Litigation Authority (now called NHS Resolution) from 250 cases between 2000 to 2010 costing over £100 million(4).

Fetal macrosomia is a well described risk factor for shoulder dystocia(5). This is variably defined as a neonatal birthweight >4.0Kg or 4.5Kg, or >90th customised or non-customised fetal weight centile. Preventative measures start with antenatal awareness of risk factors including fetal growth and size, maternal obesity and diabetes.

Earlier delivery is likely to reduce the birthweight of the infant and mitigate the main risk factor for shoulder dystocia. However, it is uncertain whether this strategy would work to reduce shoulder dystocia and its associated complications, and what effect this might have on caesarean section rates and maternal complications after delivery. Research into prevention by induction is timely, in light of conflicting messages. The Royal College of Obstetricians and Gynaecologists (RCOG) does not currently recommend induction of labour for women with a suspected macrosomic fetus in the absence of diabetes(6). However, two systematic reviews and meta-analyses found that induction of labour reduced the risk of shoulder dystocia in women who had a macrosomic fetus(7, 8). Both reviews were largely based upon the 2015 randomised controlled trial by Boulvain and colleagues of 822 pregnancies with a fetus with an estimated weight greater than the 95th centile(9). While inducing labour may reduce the risk of shoulder dystocia, it has not been shown to decrease adverse neonatal sequelae and induction is associated with a longer, more painful labour and increased risk of operative delivery(10).

The management of large-for-gestational age and macrosomic pregnancies in obstetrics was the focus of a landmark legal case heard by the UK Supreme Court in 2014(11). Mrs

For peer review only - http://bmjopen.bmj.com/site/about/guidelines.xhtml

Montgomery had type 1 diabetes and had a macrosomic baby, she was concerned about delivering her baby vaginally, but was not adequately informed of the risk of shoulder dystocia. During the delivery, shoulder dystocia occurred leading to a 12-minute delay in delivering the infant's body. Her son suffered from hypoxic ischaemic encephalopathy. A case was made that as Mrs Montgomery was not adequately informed of the risk of shoulder dystocia and its associated complications, and the alternative modes of delivery, namely caesarean section, she could not make a well-informed decision about the delivery of her son, therefore there was negligence in consent. After failed appeals at the Court of Session and the Inner house the case was finally heard at the UK Supreme court. The Supreme Court judgment in this case highlighted the obligation of clinicians to explain the risks and benefits of all treatment options, including that of no treatment, to women in order for them give a valid consent. It is therefore imperative to have robust evidence from randomised controlled trials on which to base these discussions. An investigation into the value of induction to reduce the incidence of shoulder dystocia in women with a suspected macrosomic fetus will give women and clinicians the information they need in planning their mode of delivery.

The research question is 'does induction of labour at 38^{+0} to 38^{+4} weeks' gestation, in pregnancies with suspected large-for-gestational age fetuses, reduce the incidence of shoulder dystocia?'.

This manuscript describes the trial design, setting, participants and recruitment, the intervention and control groups, randomisation, outcome measures, sample size, ethical considerations and dissemination. A separate manuscript will detail the statistical analysis plan, trial process evaluation and health economic analysis plan.

For peer review only - http://bmjopen.bmj.com/site/about/guidelines.xhtml

STUDY OBJECTIVES

Primary Objective

The primary objective is to determine the effectiveness of induction of labour at 38^{+0} to 38^{+4} weeks' gestation in reducing the incidence of shoulder dystocia in suspected large-for-gestational age fetuses.

Secondary Objective

Secondary objectives are to collect comparative data on intrapartum, perinatal, infant, maternal obstetric and long-term maternal outcomes. We will collect comparative data on maternal perceptions of their labour/birth care and physical and psychological health at two and six months postnatally.

We will report composite outcomes for intrapartum birth injury, prematurity associated problems and maternal intrapartum complication.

For peer review only

METHODS AND ANALYSIS

This protocol manuscript was written in concordance with the SPIRIT guidelines (12).

Trial Design

The Big Baby Trial is a multicentre, prospective, individually randomised controlled trial of induction of labour at 38^{+0} to 38^{+4} weeks' gestation versus standard care of fetuses that are large-for-gestational age according to ultrasound scan at 35^{+0} to 38^{+0} weeks' gestation. Our definition of large-for-gestational age is an estimated fetal weight >90th customised fetal weight centile using the woman's own customised Gestation Related Optimal Weight (GROW) chart(13). These charts provide the standard for assessment of fetal growth and newborn size, are recommended by RCOG Green Top Guidelines(14) and are in use in approximately 76% of NHS Trusts and Health Boards. The GROW 90th customised centile identifies more babies at risk of adverse outcomes than large-for-gestational age by conventional standards(15-18). Furthermore, GROW has been shown to be a better predictor of shoulder dystocia than the UK-WHO birthweight standard(19).

There is a parallel cohort study for women who decline randomisation, but wish to participate in research. This cohort includes two sub-groups. The first is women who request a planned caesarean section. The second is women who request to be delivered by early induction of labour or expectant management. The primary objective of the cohort study is to provide comparative data on those who choose planned caesarean section and confirm generalisability of the baseline data and primary outcome with the main trial.

The trial is conducted and managed by the Warwick Clinical Trials Unit and sponsored by the University Hospitals Coventry and Warwickshire NHS Trust. Funding is provided by the National Institute for Health Research (NIHR) following a commissioned call from the Health Technology Assessment Programme (HTA study reference 16/77/02). The trial is being conducted in accordance with the principals of the Declaration of Helsinki and Good Clinical Practice.

Trial Setting

For peer review only - http://bmjopen.bmj.com/site/about/guidelines.xhtml

Although we initially planned to recruit from 60 NHS Trusts over the course of the trial to enable us to enhance recruitment, this approach has changed. We now aim to recruit 80 NHS Trusts across the UK that use customised GROW charts. Staff participating in the trial must demonstrate and document a willingness to comply with the protocol, the principles of Good Clinical Practice and regulatory requirements. Furthermore, they must be prepared to participate in training and adhere to the protocol.

Participants and Recruitment

Inclusion Criteria

The study participants are women aged ≥18 years with a fetus above the 90th customised GROW fetal weight centile on ultrasound scan at 35^{+0} to 38^{+0} weeks' gestation with a cephalic presentation.

Exclusion Criteria

Box 1 lists the exclusion criteria for the study.

Box 1 – Exclusion Criteria

Exclusion Criteria
Multiple pregnancy
Pregnancy with a breech or transverse lie position
Contra-indication to induction of labour
A fetus with a known serious abnormality
A home birth or elective caesarean section already planned
A caesarean section or induction indicated due to other health conditions such as cardiac disease or hypertensive disorders
Women taking medications and/or insulin therapy for diabetes or gestational diabetes (women with these conditions who are not taking medication are eligible)
A current diagnosis of a major psychiatric disorder requiring antipsychotic medication
A previous stillbirth or neonatal death ≤28days
A current intrauterine fetal death
Prisoners

For peer review only - http://bmjopen.bmj.com/site/about/guidelines.xhtml

For peer review only

Women unable to give informed consent e.g. learning or communication difficulties that prevent the understanding of the information provided

Recruitment

Women are identified based on an ultrasound scan, performed either as part of serial fetal growth assessment or for a different indication. If the fetus has an estimated fetal weight >90th customised centile from 28^{+0} – 38^{+0} weeks' gestation, the woman can be approached and offered information about the study. Women are informed of the risks and benefits of participating and the possible risks and benefits of other delivery options. The participant information sheet and participant consent form have been assessed for clarity by the Plain English Campaign and a Crystal Mark obtained for these. By approaching women from 28^{+0} weeks' gestation, they have time to consider their participation, ask questions to health care professionals and discuss the trial with their family and friends.

The obstetrician, or consultant midwife in charge of the woman's care is asked to provide 'obstetric confirmation', to confirm they agree for their patient to participate in the trial and receive either induction of labour or standard care. This confirmation must be completed before randomisation. To be eligible a confirmatory ultrasound scan must be performed between 35^{+0} – 38^{+0} weeks' gestation. If the fetus has an estimated fetal weight >90th customised GROW centile during this gestation interval and fulfils the other eligibility criteria, the woman can participate in the trial.

Intervention and Control

Intervention

Data from the West Midlands Perinatal Episode Electronic Record (PEER) database of 161,936 pregnancies found that the median length of pregnancy for large for gestational age fetuses was 39^{+4} weeks' gestation (277 days). We further ascertained that the weekly increment of fetal weight gain in large-for-gestational age pregnancies is approximately 200g. In the trial conducted by Boulvain and colleagues, the difference in fetal weight between the induction and expectant management groups was 287g(9). Based on this, we expect that for a difference of 300g between the intervention and control arms, an interval of 1.5 weeks is required. Therefore, the intervention window for induction of labour is set at 38^{+0} to 38^{+4}

For peer review only - http://bmjopen.bmj.com/site/about/guidelines.xhtml

weeks' (266-270 days) gestation. This will ensure an approximate average of eleven days separation in gestation days between groups. Induction prior to this window may decrease the risk of shoulder dystocia but would increase the risk of neonatal complications(20-22). The method of induction is by the usual practice at the participating site Trust.

Control

The control is standard care.

Outcome Measures

Primary Outcome

The primary outcome measure is the incidence of shoulder dystocia, defined by the RCOG as 'a vaginal cephalic delivery that requires additional obstetric manoeuvres to deliver the fetus after the head has delivered and gentle traction has failed'(6). These data are being extracted from clinical notes.

As the sites are unblinded, all delivery notes are reviewed by an independent expert panel to confirm if shoulder dystocia has occurred. The independent panel consists of a senior obstetrician, a senior neonatologist, a senior midwife and a trainee obstetrician. Delivery notes are anonymised. The independent panel is blind to the trial allocation. Two panel members review each set of notes and categorise the notes into: 1. delivered by caesarean section; 2. no shoulder dystocia; 3. shoulder dystocia; or 4. needs more clarification. Where more clarification is needed, additional information is being sought from trial sites. If there is discrepancy between panel members, the entire panel discusses the case until a consensus decision is made.

Secondary Outcomes

The secondary outcomes are grouped into maternal peripartum, fetal peripartum, neonatal outcomes and longer-term outcomes. The secondary outcomes captured from the admission for delivery are defined in Box 2.

Box 2. Secondary Outcomes

For peer review only - http://bmjopen.bmj.com/site/about/guidelines.xhtml

For peer review only

Maternal Peripartum	Fetal Peripartum	Neonatal
Duration of hospital stay prior to delivery	Time recorded between delivery of the head and delivery of the body	Stillbirth
Duration of hospital stay after delivery	Time in labour ward	Neonatal death
Mode of delivery	Time from commencement of the active second stage of labour until fetal expulsion	Birthweight
Perineal tears		Gestation at birth
Vaginal and cervical lacerations		Apgar score at five minutes
Primary postpartum haemorrhage		Fractures
Clinician defined sepsis		Brachial plexus injury
Fever >38.0°C given antibiotics		Clinician defined sepsis
Retained placenta		Given antibiotics
Uptake of breastfeeding		Admission to the neonatal unit (intensive, special or transitional care)
Hospital readmission within 30 days of postnatal inpatient discharge		Duration of hospital stay
Death		Hypoxic ischaemic encephalopathy
		Use of phototherapy
		Respiratory morbidity
		Hypoglycaemia

Randomised participants and participants in the cohort study opting for an elective caesarean section are asked to complete questionnaires at two and six months postpartum. The outcomes for the infants are assessed according to the proportion under specialist medical care at two months for a problem related to intrapartum experience, maternal report of infant health concerns at six months, in hospital healthcare costs and hospital readmission within 30 days of postnatal inpatient discharge. Responses from these questionnaires identify infants who have sustained a potential birth-related injury. Relevant data related to the injury are being requested from sites and an independent adjudication committee will classify these as delivery / not delivery related. This will be undertaken by the same independent

For peer review only

adjudication committee that is to review the delivery notes. Box 3 details the longer-term maternal and neonatal outcomes.

Box 3. Longer-term Maternal and Neonatal Outcomes

**Longer-term Outcomes**
Maternal experience (six simple questions) at two months(23)
Duration of exclusive breastfeeding at two and six months
Health-related quality of life (EQ-5D-5L) at two and six months(24)
Edinburgh Postnatal Depression Scale score at two and six months(25)
Impact of Events Scale at two months(26)
Postpartum bonding questionnaire at two months(27)
Maternal report of infant health at two and six months
Urinary incontinence ICIQ-UI short form at two and six months(28)
Faecal incontinence at two and six months
Sexual function at six months
Maternal and infant death at six months from HES-ONS linked mortality data
Participants health resource used for the economic analysis for mother and baby at two and six months

The three composite outcomes are:

1. Peripartum birth injury - includes one or both of fractured or brachial plexus injury.
2. Prematurity associated problems which include one or more of phototherapy, clinician defined sepsis before discharge from hospital, or respiratory support
3. Maternal peripartum complications which include one or more of 3rd and 4th degree perineal tears, vaginal/cervical lacerations, clinician defined sepsis before discharge from hospital or primary postpartum haemorrhage.

Sample Size

The true incidence of shoulder dystocia in women with a fetus >90th customised GROW centile is unknown. In the trial by Boulvain and colleagues on suspected macrosomia, the incidence of shoulder dystocia, defined as ‘difficulty with delivery of the shoulders not resolved by McRoberts manoeuvre’, in the control arm was 16/411 (3.9%)(9). In the Big Baby Trial, we have used a similar definition of shoulder dystocia, and have estimated the incidence of shoulder dystocia in the control group to be 4%. Boulvain et al. found a relative risk for significant shoulder dystocia in the intervention group to be 0.32 (95% CI 0.12-0.85)(9). Considering this, we have set the effect size to 50% reduction in the primary outcome to 2%.

For peer review only - http://bmjopen.bmj.com/site/about/guidelines.xhtml

This reduction is considered clinically worthwhile. To achieve a 50% reduction in the primary outcome at a 5% significance level with 90% power, 1,626 women would need to be allocated to each arm, with a sample size of 3252 women.

The sample size for this trial has been increased from 3,252 by 23% to 4,000. This is to allow for some women giving birth prior to the intervention, and to account for uncertainty in the event rate in the control group. In the trial by Boulvain and colleagues, 31/408 women (7.6%) gave birth prior to the intervention(9). The increase in the sample size also takes into account the unknown incidence of the primary outcome, an expected small loss of primary outcome, and any effect of clustering at site - although an unpublished analysis of national Growth Assessment Protocol (GAP) data by the Perinatal Institute indicated the intra-cluster correlation coefficient for being large-for-gestational age to be <0.00055, suggesting that any effect will be negligible.

The trial Data Monitoring and Ethics Committee are presented with a closed and open report of the data every six months of the study. A key event analysis was undertaken once primary outcome data were collected for 1,000 participants, given the uncertainty in the sample size estimate. The Data Monitoring and Ethics Committee was asked to advise if a sample size adjustment was required based upon the incidence of shoulder dystocia in the control arm. These data were available on the 5th February 2020 and were considered by the Data Monitoring and Ethics Committee who were unanimous in their satisfaction of the original planned target and recommended that the trial continues to recruit the planned 4,000 women.

Internal Pilot, Process Evaluation and Qualitative Interviews

Recruitment was assessed when ten sites had been recruiting for three months. A formative process evaluation was undertaken to assess barriers to recruitment of sites and participants and barriers to follow-up. This included interviews with ten clinicians to explore adherence to study protocol, impact on workload and impact of the trial on the woman's decision-making process. Feedback from the pilot study and process evaluation allowed us to run seamlessly into the main study. This will be described in a further manuscript.

For peer review only - http://bmjopen.bmj.com/site/about/guidelines.xhtml

Randomisation

Randomisation is provided by Warwick Clinical Trials Unit using an online web application or telephone. Women are randomised using minimisation, balancing site, fetal weight centile (≤95th or >95th estimated fetal weight centile) and maternal age (≤35 or >35 years of age). To ensure allocation concealment, randomisation only takes place once all the baseline data have been collected. Women are randomised to either booking of induction of labour between 38^{+0}-38^{+4} weeks' gestation or to standard care. Women are immediately informed of the allocation.

Data Collection

Anonymised data are entered into a secured password protected trial database, developed by the programming team at Warwick Clinical Trials Unit, either at site or by the Warwick Clinical Trials Unit. Participants are identified by a unique study number. All data are stored securely and held in accordance with the relevant UK data protection legislation.

The baseline data collected are maternal height, weight, age, parity, ethnic origin, previous obstetric history, current obstetric history, tobacco use and use of antenatal corticosteroids. Women are asked to complete the EQ-5D-5L health-related quality of life questionnaire(24), Edinburgh Postnatal Depression Scale score(25), urinary incontinence ICIQ-UI short form(28), and questions on faecal incontinence and sexual function at baseline.

The fetal and neonatal outcomes collected are detailed in Box 2. In addition, we are collecting data on the proportion of infants under specialist medical care at two months for a problem related to intrapartum experience, a maternal report of infant health at six months and in-hospital costs. The maternal outcomes collected are described in Box 2. Longer-term maternal outcomes to be collected are described in Box 3.

Follow-up questionnaires are sent to participants at two- and six-months postpartum. We check the hospital electronic record for notification of a neonatal death in all infants participating in the study who were discharged home, prior to sending the follow-up questionnaires. All study related data are stored in accordance with all applicable regulatory requirements and access is restricted to authorised personnel. Trial records and associated

For peer review only

documentation will be archived for 25 years for the randomised participants and ten years for the cohort participants.

For the parallel cohort we collect the same baseline data as the randomised controlled trial. For women requesting a planned caesarean section we collect the same maternal, neonatal and infant outcomes as the randomised controlled trial. There is a limited data collection for women in the cohort study who request induction or standard care. Women have been consented to be approached for longer-term follow up.

Data Analysis

All analyses will be by intention to treat at the time of randomisation. The primary analysis will compare the incidence of shoulder dystocia between the intervention and control groups. The comparison will be made using logistic regression models both unadjusted and adjusted for appropriate covariates. Other secondary binary outcomes will be assessed in a similar way. Continuous outcomes will be analysed using linear regression models; both adjusted and unadjusted analyses will be computed. A description of the data analyses are described in a further manuscript.

For peer review only

ETHICS AND DISSEMINATION

Ethical Conduct of the Trial

The trial complies with all UK legislation and Warwick Clinical Trials Unit standard operating procedures. Health Research Authority approval and NHS Trust R&D approval was obtained before participants were enrolled in the trial. The trial's International Standard Randomised Controlled Trial number is 18229892.

A key ethical challenge in this trial was to ensure that robust informed consent was obtained from participants. The trial requires women to consent to being randomised to a specific management pathway for the birth of their child rather than the standard clinical practice of a shared decision-making process with their clinician. It was therefore an imperative to provide the best possible information to women about the risks and benefits of all management options so they could make an informed decision about trial participation in the wider context of decision-making about their clinical care. In developing our information materials and consent processes we were guided by the standard set by the Supreme Court judgment in Montgomery(11). The key steps we took to develop the information and consent processes were:

- A review of all relevant literature from the RCOG, National Institute for Health and Care Excellence and other published works.
- Development of participants facing materials with the patient and public involvement representatives.
- A thorough peer review obstetricians of all participant facing materials.
- The inclusion of a cohort group to respect the woman's preferred choice.

Adverse Event Management

Adverse events are being collected from the time of randomisation until delivery. Serious adverse events are being collected from the time of randomisation until 30 days after initial discharge following delivery. Adverse events and serious adverse events are being identified when collecting outcome data or when completing the two-month follow-up questionnaires.

For peer review only - http://bmjopen.bmj.com/site/about/guidelines.xhtml

For the trial only, adverse events affecting the woman or her baby which could be potentially related to the pregnancy, delivery or care of the neonate are being collected. Adverse events are being collected for all participants in the randomised controlled trial and participants in the cohort study requesting an elective caesarean section.

Serious adverse events are only being collected for participants in the randomised controlled trial and need to be reported to Warwick Clinical Trials Unit within 24 hours of the site being made aware of the event. Certain events that would meet the definition of serious adverse events are common in pregnancy and for this trial do not need to be reported as serious adverse events. These events are being reported in the trial case report forms and comparative rates will be monitored by the data monitoring and ethics committee. Serious adverse events that require immediate reporting for the woman and neonate are described in Box 4.

Box 4: Serious adverse events that require immediate reporting for the woman and neonate

**Maternal Serious Adverse Events**	**Neonatal Serious Adverse Events**
Maternal death	Stillbirth
Inpatient admission to intensive care and/or high dependency unit at any time during pregnancy/postnatal period	Infant death
Readmission to hospital within 30 days of initial postnatal discharge	Inpatient admission to the neonatal unit
Antenatal hospital admission not related to pregnancy	Inpatient readmission to hospital within 30 days of initial postnatal discharge*
Transfer out of the maternity unit for further inpatient care	
Inpatient admission to a mental health unit	
Symphysiotomy	

*Except for respiratory tract infection, jaundice, urinary tract infection, weight loss lasting less than 5 days, reflux and constipation.

For all serious adverse events a clinical assessment of causality is being made by a medical doctor as to whether the event is related to the booking of induction of labour. If the site or sponsor determine that there is a possible, probable, or definite relationship to the intervention then an assessment of expectedness is completed. Related and unexpected serious adverse events are expedited to the Health Research Authority Research Ethics

For peer review only - http://bmjopen.bmj.com/site/about/guidelines.xhtml

Committee, the sponsor and the chairs of the Trial Steering Committee and Data Monitoring and Ethics Committee.

Monitoring

All clinicians involved in obtaining consent are required to have completed Good Clinical Practice training. A programme of training is being delivered to all staff participating in the trial at site level. Data entered onto the trial database are being checked for accuracy and completeness by Warwick Clinical Trials Unit in accordance with the trial data management plan. A risk assessment is being undertaken and forms the basis of the trial monitoring plan. Following site initiation, the trial team is in regular contact with sites.

Patient and Public Involvement

Karen Hillyer (Chair) and Jackie Dewdney (Board Member) of the Erb's Palsy group are actively involved in the planning and development of this trial. The Erb's Palsy group is a UK-based not for profit organisation which offers advice, support and information to families affected by Erb's Palsy. Karen and Jackie led on the development of all patient-facing materials. As co-applicants they are involved in all aspects of the trial and will help inform the interpretation of the final results and dissemination of findings.

Progress so far

The trial started recruiting on 8th June 2018. As of 17th September 2021, there are 2261 randomised participants and 1566 cohort participants. Recruitment was paused on the 23rd March 2020 because of the COVID-19 pandemic. This restarted on a site-by-site basis depending on site capacity from 22nd May 2020.

Dissemination

The trial results will be reported in the NIHR journals library and published in an open access peer reviewed journal. Findings will be made available on the University of Warwick and Perinatal Institute websites. Abstracts will be submitted to major national and international conferences. Three dissemination events will be held for key stakeholders at the end of the trial. The trial will be reported in accordance with CONSORT guidelines. All publications will be submitted to the NIHR-HTA Programme for approval prior to submission for publication.

For peer review only - http://bmjopen.bmj.com/site/about/guidelines.xhtml

CHANGES MADE SINCE FUNDING AGREED

Since submission of the detailed project description to the NIHR-HTA some changes have been made to the protocol and agreed by the Trial Steering Committee, and Data Monitoring and Ethics Committee. This section details the changes made and reasons for these.

Initially we predicted we would need 60 sites to reach our recruitment target. Over the course of the trial, it was evident this would need to be increased to 80 sites to enable us to improve recruitment and reach our target of 4,000 women randomised in a timely manner. In the application to the NIHR-HTA we wanted to collect outcomes on women in the cohort study who had requested an elective caesarean section. It was decided by the Trial Management Group and Trial Steering Committee that this should be extended to include outcomes on women who decline randomisation but chose either to have an early induction of labour or expectant management. The objective of this group was to provide comparative data on those who choose the timing of the birth and to confirm generalisability of the baseline data and primary outcome. Women with a current intrauterine fetal death were added to the current exclusion criteria as it is inappropriate to randomise these women and different plans would be made regarding their delivery. Prisoners were also added as a new exclusion criterion as there is a different ethical framework for their participation in medical research.

In the initial application to the NIHR-HTA we suggested that SAEs will be reported for any incidences of stillbirth, maternal death, serious intrapartum injury to the fetus or any other event that could be classified with similar severity. Once the trial had started recruiting a substantial number of SAEs were being reported that were classified as outcomes for the trial. Therefore, more formal guidance was formulated to avoid repetition in the data collection for events that did not meet the definition of SAE and to give clear instructions to the sites about what needed to be reported.

As a consequence of ongoing COVID -19 risk we are implementing a new consent process to allow for remote electronic consent rather than all consent being taken in person.

For peer review only - http://bmjopen.bmj.com/site/about/guidelines.xhtml

REFERENCES

1. Ahn ES, Jung MS, Lee YK, Ko SY, Shin SM, Hahn MH. Neonatal clavicular fracture: recent 10 year study. Pediatr Int. 2015;57(1):60-3.
2. Chauhan SP, Blackwell SB, Ananth CV. Neonatal brachial plexus palsy: incidence, prevalence, and temporal trends. Semin Perinatol. 2014;38(4):210-8.
3. Iffy L, Brimacombe M, Apuzzio JJ, Varadi V, Portuondo N, Nagy B. The risk of shoulder dystocia related permanent fetal injury in relation to birth weight. European Journal of Obstetrics & Gynecology and Reproductive Biology. 2008;136(1):53-60.
4. Anderson A. Ten Years of maternity claims: an analysis of the NHS Litigation Authority data - key findings. Clinical Risk. 2013;19(1):24-31.
5. Hansen A, Chauhan SP. Shoulder dystocia: definitions and incidence. Semin Perinatol. 2014;38(4):184-8.
6. Royal College of Obstetricians and Gynaecologists. Green-top Guideline No. 42 Shoulder Dystocia. 2nd ed. London2012.
7. Boulvain M, Irion O, Dowswell T, Thornton JG. Induction of labour at or near term for suspected fetal macrosomia. Cochrane Database Syst Rev. 2016(5):CD000938.
8. Magro-Malosso ER, Saccone G, Chen M, Navathe R, Di Tommaso M, Berghella V. Induction of labour for suspected macrosomia at term in non-diabetic women: a systematic review and meta-analysis of randomized controlled trials. Bjog. 2017;124(3):414-21.
9. Boulvain M, Senat MV, Perrotin F, Winer N, Beucher G, Subtil D, et al. Induction of labour versus expectant management for large-for-date fetuses: a randomised controlled trial. Lancet. 2015;385(9987):2600-5.
10. Middleton P, Shepherd E, Crowther CA. Induction of labour for improving birth outcomes for women at or beyond term. Cochrane Database Syst Rev. 2018;5:CD004945.
11. Montgomery (Appelant) vs Lanarkshire Health Board (Respondant). Montgomery (Appelant) vs Lanarkshire Health Board (Respondant),: Supreme Court; 2015.
12. Chan AW, Tetzlaff JM, Altman DG, Laupacis A, Gotzsche PC, Krleza-Jeric K, et al. SPIRIT 2013 statement: defining standard protocol items for clinical trials. Ann Intern Med. 2013;158(3):200-7.
13. Perinatal Institute. Gestation Network Perinatal Institute2020 [Available from: https://www.gestation.net.
14. Royal College of Obstetricians and Gynaecologists. Green-top Guideline No. 31 The Investigation and Management of the Small-For-Gestational-Age Fetus. 2nd ed. London: Royal College of Obstetricians and Gynaecologists; 2013.
15. Larkin JC, Speer PD, Simhan HN. A customized standard of large size for gestational age to predict intrapartum morbidity. Am J Obstet Gynecol. 2011;204(6):499 e1-10.
16. Cha HH, Kim JY, Choi SJ, Oh SY, Roh CR, Kim JH. Can a customized standard for large for gestational age identify women at risk of operative delivery and shoulder dystocia? J Perinat Med. 2012;40(5):483-8.
17. Pasupathy D, McCowan LM, Poston L, Kenny LC, Dekker GA, North RA, et al. Perinatal outcomes in large infants using customised birthweight centiles and conventional measures of high birthweight. Paediatr Perinat Epidemiol. 2012;26(6):543-52.
18. Sjaarda LA, Albert PS, Mumford SL, Hinkle SN, Mendola P, Laughon SK. Customized large-for-gestational-age birthweight at term and the association with adverse perinatal outcomes. Am J Obstet Gynecol. 2014;210(1):63 e1- e11.
19. Norris T, Johnson W, Farrar D, Tuffnell D, Wright J, Cameron N. Small-for-gestational age and large-for-gestational age thresholds to predict infants at risk of adverse delivery and

For peer review only - http://bmjopen.bmj.com/site/about/guidelines.xhtml

neonatal outcomes: are current charts adequate? An observational study from the Born in Bradford cohort. BMJ Open. 2015;5(3):e006743.
20. Engle WA. Morbidity and mortality in late preterm and early term newborns: a continuum. Clin Perinatol. 2011;38(3):493-516.
21. Dietz PM, Rizzo JH, England LJ, Callaghan WM, Vesco KK, Bruce FC, et al. Early term delivery and health care utilization in the first year of life. J Pediatr. 2012;161(2):234-9 e1.
22. Parikh LI, Reddy UM, Mannisto T, Mendola P, Sjaarda L, Hinkle S, et al. Neonatal outcomes in early term birth. Am J Obstet Gynecol. 2014;211(3):265 e1- e11.
23. Harvey S, Rach D, Stainton MC, Jarrell J, Brant R. Evaluation of satisfaction with midwifery care. Midwifery. 2002;18(4):260-7.
24. Gusi N, Olivares PR, Rajendram R. The EQ-5D Health-Related Quality of Life Questionnaire. In: Preedy V, Watson R, editors. Handbook of Disease Burdens and Quality of Life Measures. New York: Springer; 2010. p. 87-99.
25. Cox JL, Holden JM, Sagovsky R. Detection of postnatal depression. Development of the 10-item Edinburgh Postnatal Depression Scale. Br J Psychiatry. 1987;150:782-6.
26. Horowitz M, Wilner N, Alvarez W. Impact of Event Scale: a measure of subjective stress. Psychosom Med. 1979;41(3):209-18.
27. Taylor A, Atkins R, Kumar R, Adams D, Glover V. A new Mother-to-Infant Bonding Scale: links with early maternal mood. Arch Womens Ment Health. 2005;8(1):45-51.
28. Avery K, Donovan J, Peters TJ, Shaw C, Gotoh M, Abrams P. ICIQ: a brief and robust measure for evaluating the symptoms and impact of urinary incontinence. Neurourol Urodyn. 2004;23(4):322-30.

For peer review only

AUTHOR CONTRIBUTIONS

All authors read and approved the manuscript. All authors have contributed to the study design. SQ and JG are the Co-chief Investigators and oversee the running of the study. MU input into all aspects of the study design and support in running the study. LE is a Clinical Research Fellow and assisted with all aspects of the delivery of the interventions at site level. SW, KH, RG and JB managed the trial and data management. DB, EB, KF, SD, AG provided the clinician and midwifery input into the study. JF carried out the process evaluation. KB, RL and SG were the statisticians for the study. JD, KH were the Patient and Public Involvement representatives. SP and HM provided oversight of the health economic aspects of the study. A-MS was the ethicist for the study. AW and MW over sited the programming and database management and CJ was the sponsorship representative.

For peer review only

FUNDING STATEMENT

This project is funded by the National Institute for Health Research, Health Technology Assessment (NIHR HTA) project number 16/77/02. The views and opinions expressed therein are those of the authors and do not necessarily reflect those of the HTA, NIHR, NHS or the Department of Health.

For peer review only

COMPETING INTERESTS STATEMENT

JG is the director of the Perinatal Institute, a not for profit organisation, limited by guarantee, and a qualified provider of maternity support services to the NHS. It derives its income from some of its products and services, including the award-winning GAP program mentioned in this protocol, through which they have been able to implement training, e-learning and protocols in the majority of Trusts and Health Boards in the UK. GAP includes the standardised, RCOG endorsed customised GROW charts which will be used to identify large-for-gestational age as the entry point for this trial.

MU is chief investigator or co-investigator on multiple previous and current research grants from the UK National Institute for Health Research, Arthritis Research UK and is a co-investigator on grants funded by the Australian NHMRC. He is an NIHR Senior Investigator. He has received travel expenses for speaking at conferences from the professional organisations hosting the conferences. He is a director and shareholder of Clinvivo Ltd http://www.clinvivo.com that provides electronic data collection for health services research. He is part of an academic partnership with Serco Ltd, funded by the European Social Fund, related to return to work initiatives. He is a co-investigator on two NIHR funded studies receiving additional support from Stryker Ltd. He has accepted honoraria for teaching/lecturing from consortium for advanced research training in Africa. He was until March 2020 an editor of the NIHR journal series, and a member of the NIHR Journal Editors Group, for which he received a fee.

For peer review only

WORD COUNT

4156

For peer review only

For peer review only - http://bmjopen.bmj.com/site/about/guidelines.xhtml

For peer review only

For peer review only - http://bmjopen.bmj.com/site/about/guidelines.xhtml

SPIRIT

STANDARD PROTOCOL ITEMS: RECOMMENDATIONS FOR INTERVENTIONAL TRIALS

SPIRIT 2013 Checklist: Recommended items to address in a clinical trial protocol and related documents*

Section/item	ItemNo	Description	Page found
**Administrative information**			
Title	1	Descriptive title identifying the study design, population, interventions, and, if applicable, trial acronym	1
Trial registration	2a	Trial identifier and registry name. If not yet registered, name of intended registry	2
	2b	All items from the World Health Organization Trial Registration Data Set	2
Protocol version	3	Date and version identifier	n/a
Funding	4	Sources and types of financial, material, and other support	7,23
Roles and responsibilities	5a	Names, affiliations, and roles of protocol contributors	1,22
	5b	Name and contact information for the trial sponsor	7,22
	5c	Role of study sponsor and funders, if any, in study design; collection, management, analysis, and interpretation of data; writing of the report; and the decision to submit the report for publication, including whether they will have ultimate authority over any of these activities	7
	5d	Composition, roles, and responsibilities of the coordinating centre, steering committee, endpoint adjudication committee, data management team, and other individuals or groups overseeing the trial, if applicable (see Item 21a for data monitoring committee)	throughout
**Introduction**			
Background and rationale	6a	Description of research question and justification for undertaking the trial, including summary of relevant studies (published and unpublished) examining benefits and harms for each intervention	5
	6b	Explanation for choice of comparators	9
Objectives	7	Specific objectives or hypotheses	5

For peer review only - http://bmjopen.bmj.com/site/about/guidelines.xhtml

Trial design	8	Description of trial design including type of trial (eg, parallel group, crossover, factorial, single group), allocation ratio, and framework (eg, superiority, equivalence, noninferiority, exploratory)	7

Methods: Participants, interventions, and outcomes

Study setting	9	Description of study settings (eg, community clinic, academic hospital) and list of countries where data will be collected. Reference to where list of study sites can be obtained	7
Eligibility criteria	10	Inclusion and exclusion criteria for participants. If applicable, eligibility criteria for study centres and individuals who will perform the interventions (eg, surgeons, psychotherapists)	8
Interventio ns	11a	Interventions for each group with sufficient detail to allow replication, including how and when they will be administered	9
	11b	Criteria for discontinuing or modifying allocated interventions for a given trial participant (eg, drug dose change in response to harms, participant request, or improving/worsening disease)	9
	11c	Strategies to improve adherence to intervention protocols, and any procedures for monitoring adherence (eg, drug tablet return, laboratory tests)	n/a
	11d	Relevant concomitant care and interventions that are permitted or prohibited during the trial	n/a
Outcomes	12	Primary, secondary, and other outcomes, including the specific measurement variable (eg, systolic blood pressure), analysis metric (eg, change from baseline, final value, time to event), method of aggregation (eg, median, proportion), and time point for each outcome. Explanation of the clinical relevance of chosen efficacy and harm outcomes is strongly recommended	10
Participant timeline	13	Time schedule of enrolment, interventions (including any run-ins and washouts), assessments, and visits for participants. A schematic diagram is highly recommended (see Figure)	9
Sample size	14	Estimated number of participants needed to achieve study objectives and how it was determined, including clinical and statistical assumptions supporting any sample size calculations	12
Recruitme nt	15	Strategies for achieving adequate participant enrolment to reach target sample size	13

Methods: Assignment of interventions (for controlled trials)

Allocation:

For peer review only - http://bmjopen.bmj.com/site/about/guidelines.xhtml

Sequence generation	16a	Method of generating the allocation sequence (eg, computer-generated random numbers), and list of any factors for stratification. To reduce predictability of a random sequence, details of any planned restriction (eg, blocking) should be provided in a separate document that is unavailable to those who enrol participants or assign interventions	14
Allocation concealment mechanism	16b	Mechanism of implementing the allocation sequence (eg, central telephone; sequentially numbered, opaque, sealed envelopes), describing any steps to conceal the sequence until interventions are assigned	14
Implementation	16c	Who will generate the allocation sequence, who will enrol participants, and who will assign participants to interventions	14
Blinding (masking)	17a	Who will be blinded after assignment to interventions (eg, trial participants, care providers, outcome assessors, data analysts), and how	14
	17b	If blinded, circumstances under which unblinding is permissible, and procedure for revealing a participant's allocated intervention during the trial	n/a
**Methods: Data collection, management, and analysis**			
Data collection methods	18a	Plans for assessment and collection of outcome, baseline, and other trial data, including any related processes to promote data quality (eg, duplicate measurements, training of assessors) and a description of study instruments (eg, questionnaires, laboratory tests) along with their reliability and validity, if known. Reference to where data collection forms can be found, if not in the protocol	14
	18b	Plans to promote participant retention and complete follow-up, including list of any outcome data to be collected for participants who discontinue or deviate from intervention protocols	14
Data management	19	Plans for data entry, coding, security, and storage, including any related processes to promote data quality (eg, double data entry; range checks for data values). Reference to where details of data management procedures can be found, if not in the protocol	14
Statistical methods	20a	Statistical methods for analysing primary and secondary outcomes. Reference to where other details of the statistical analysis plan can be found, if not in the protocol	14
	20b	Methods for any additional analyses (eg, subgroup and adjusted analyses)	14
	20c	Definition of analysis population relating to protocol non-adherence (eg, as randomised analysis), and any statistical methods to handle missing data (eg, multiple imputation)	14
**Methods: Monitoring**			

Data monitoring	21a	Composition of data monitoring committee (DMC); summary of its role and reporting structure; statement of whether it is independent from the sponsor and competing interests; and reference to where further details about its charter can be found, if not in the protocol. Alternatively, an explanation of why a DMC is not needed	13
	21b	Description of any interim analyses and stopping guidelines, including who will have access to these interim results and make the final decision to terminate the trial	13
Harms	22	Plans for collecting, assessing, reporting, and managing solicited and spontaneously reported adverse events and other unintended effects of trial interventions or trial conduct	16
Auditing	23	Frequency and procedures for auditing trial conduct, if any, and whether the process will be independent from investigators and the sponsor	18
**Ethics and dissemination**			
Research ethics approval	24	Plans for seeking research ethics committee/institutional review board (REC/IRB) approval	16
Protocol amendments	25	Plans for communicating important protocol modifications (eg, changes to eligibility criteria, outcomes, analyses) to relevant parties (eg, investigators, REC/IRBs, trial participants, trial registries, journals, regulators)	8
Consent or assent	26a	Who will obtain informed consent or assent from potential trial participants or authorised surrogates, and how (see Item 32)	9
	26b	Additional consent provisions for collection and use of participant data and biological specimens in ancillary studies, if applicable	n/a
Confidentiality	27	How personal information about potential and enrolled participants will be collected, shared, and maintained in order to protect confidentiality before, during, and after the trial	14
Declaration of interests	28	Financial and other competing interests for principal investigators for the overall trial and each study site	24
Access to data	29	Statement of who will have access to the final trial dataset, and disclosure of contractual agreements that limit such access for investigators	in main protocol
Ancillary and post-trial care	30	Provisions, if any, for ancillary and post-trial care, and for compensation to those who suffer harm from trial participation	n/a
Dissemination policy	31a	Plans for investigators and sponsor to communicate trial results to participants, healthcare professionals, the public, and other relevant groups (eg, via publication, reporting in results databases, or other data sharing arrangements), including any publication restrictions	18

For peer review only

	31b	Authorship eligibility guidelines and any intended use of professional writers	22
	31c	Plans, if any, for granting public access to the full protocol, participant-level dataset, and statistical code	18
**Appendices**			
Informed consent materials	32	Model consent form and other related documentation given to participants and authorised surrogates	n/a
Biological specimens	33	Plans for collection, laboratory evaluation, and storage of biological specimens for genetic or molecular analysis in the current trial and for future use in ancillary studies, if applicable	n/a

*It is strongly recommended that this checklist be read in conjunction with the SPIRIT 2013 Explanation & Elaboration for important clarification on the items. Amendments to the protocol should be tracked and dated. The SPIRIT checklist is copyrighted by the SPIRIT Group under the Creative Commons "Attribution-NonCommercial-NoDerivs 3.0 Unported" license.

For peer review only

BMJ Open

Induction of Labour for Predicted Macrosomia: Study Protocol for the 'Big Baby' Randomised Controlled Trial

Journal:	*BMJ Open*
Manuscript ID	bmjopen-2021-058176.R1
Article Type:	Protocol
Date Submitted by the Author:	27-Apr-2022
Complete List of Authors:	Ewington, Lauren; University of Warwick Faculty of Medicine, Biomedical Sciences; University Hospitals Coventry and Warwickshire NHS Trust, Women's and Children's<br>Gardosi, Jason; Perinatal Institute<br>Lall, Ranjit; University of Warwick, Warwick Clinical Trials Unit<br>Underwood, Martin; Warwick University, Warwick Clinical Trials Unit; University Hospitals Coventry and Warwickshire NHS Trust<br>Fisher, Joanne; University of Warwick Warwick Medical School, Clinical Trials Unit<br>Wood, Sara; University of Warwick, Warwick Clinical Trials Unit<br>Griffin, Ryan; University of Warwick, Warwick Clinical Trials Unit<br>Harris, Kirsten; University of Warwick<br>Bick, Debra; University of Warwick, Warwick Clinical Trials Unit, Warwick Medical School<br>Booth, Katie; University of Warwick, Warwick Clinical Trials Unit<br>Brown, Jaclyn; University of Warwick, Clinical Trials Unit<br>Butler, Emily; Perinatal Institute<br>Fowler, Kelly; Perinatal Institute<br>Williams, Mandy; Perinatal Institute<br>Deshpande, Sanjeev; Shrewsbury and Telford Hospital NHS Trust<br>Gornall, Adam; Shrewsbury and Telford Hospital NHS Trust<br>Dewdney, Jackie; Erb's Palsy Group<br>Hillyer, Karen; Erb's Palsy Group<br>Gates, Simon; University of Birmingham, Cancer Research UK Clinical Trials Unit<br>Jones, Ceri; University Hospitals Coventry and Warwickshire NHS Trust<br>Mistry, Hema; University of Warwick, Warwick Clinical Trials Unit<br>Petrou, Stavros; Nuffield Department of Primary Care Health Sciences<br>Slowther, Anne-Marie; University of Warwick, Social Sciences and Systems in Health<br>Willis, Adrian; University of Warwick, Warwick Clinical Trials Unit<br>Quenby, Siobhan; University of Warwick; University Hospitals Coventry and Warwickshire NHS Trust
**Primary Subject Heading**:	Obstetrics and gynaecology
Secondary Subject Heading:	Obstetrics and gynaecology
Keywords:	OBSTETRICS, MEDICAL ETHICS, Ultrasonography < OBSTETRICS

SCHOLARONE™
Manuscripts

Induction of Labour for Predicted Macrosomia: Study Protocol for the 'Big Baby' Randomised Controlled Trial

Lauren Jade Ewington[1,2], Jason Gardosi[3], Ranjit Lall[4], Martin Underwood[2,4], Joanne Fisher[4], Sara Wood[4], Ryan Griffin[4], Kirsten Harris[4], Debra Bick[4], Katie Booth[4], Jaclyn Brown[4], Emily Butler[3], Kelly Fowler[3], Mandy Williams[3], Sanjeev Deshpande[5], Adam Gornall[5], Jackie Dewdney[6], Karen Hillyer[6], Simon Gates[7], Ceri Jones[2], Hema Mistry[4], Stavros Petrou[8], Anne-Marie Slowther[9], Adrian Willis[4], Siobhan Quenby[1,2]

Author Affiliations:

1. Division of Biomedical Sciences, University of Warwick, Coventry, UK
2. University Hospitals Coventry and Warwickshire NHS Trust, Coventry, UK
3. Perinatal Institute, Birmingham, UK
4. Warwick Clinical Trials Unit, University of Warwick, Coventry, UK
5. Shrewsbury and Telford Hospitals NHS Trust, Telford, UK
6. Erb's Palsy Group, Coventry, UK
7. Cancer Clinical Trials Unit, University of Birmingham, Birmingham, UK
8. Nuffield Department of Primary Care Health Sciences, University of Oxford, Oxford, UK
9. Social Sciences and Systems in Health, University of Warwick, Coventry, UK

Corresponding Author:

Professor Siobhan Quenby: s.quenby@warwick.ac.uk

For peer review only

For peer review only - http://bmjopen.bmj.com/site/about/guidelines.xhtml

ABSTRACT

Introduction

Large-for-gestational age (LGA) fetuses have an increased risk of shoulder dystocia. This can lead to adverse neonatal outcomes and death. Early induction of labour in women with a fetus suspected to be macrosomic may mitigate the risk of shoulder dystocia. The Big Baby Trial aims to find if induction of labour at 38^{+0}-38^{+4} weeks' gestation, in pregnancies with suspected LGA fetuses, reduces the incidence of shoulder dystocia.

Methods and Analysis

The Big Baby Trial is a multicentre, prospective, individually randomised controlled trial of induction of labour at 38^{+0} to 38^{+4} weeks' gestation versus standard care as per each hospital trust (median gestation of delivery 39^{+4}) among women whose fetuses have an estimated fetal weight >90th customised centile according to ultrasound scan at 35^{+0} to 38^{+0} weeks' gestation. There is a parallel cohort study for women who decline randomisation because they opt for induction, expectant management or caesarean section. Up to 4,000 women will be recruited and randomised to induction of labour or to standard care. The primary outcome is the incidence of shoulder dystocia; assessed by an independent expert group, blind to treatment allocation, from delivery records. Secondary outcomes include birth trauma, fractures, haemorrhage, caesarean section rate and length of inpatient stay. The main trial is ongoing, following an internal pilot study. A qualitative reporting, health economic evaluation and parallel process evaluation are included.

Ethics and Dissemination

The study received a favourable opinion from the South West – Cornwall and Plymouth Health Research Authority on 23/03/2018 (IRAS project ID 229163). Study results will be reported in the NIHR journal library and published in an open access peer reviewed journal. We will plan dissemination events for key stakeholders.

Trial Registration Number

ISRCTN18229892

For peer review only

For peer review only - http://bmjopen.bmj.com/site/about/guidelines.xhtml

STRENGHTS AND LIMITATIONS OF THIS STUDY

- This is the largest trial assessing if induction of labour decreases the incidence of shoulder dystocia in women with a suspected large-for-gestational age fetus.
- The main trial is currently open to recruitment, following a successful internal pilot study. The trial includes qualitative reporting, and health economic and process evaluations.
- Women declining randomisation and opting for an elective caesarean section can consent to participate in a parallel cohort study to collect maternal and neonatal health outcomes.
- Recruitment is challenging as women and clinicians often have a preference regarding timing and mode of birth and decline randomisation. Therefore, it is unclear if the women randomised into the trial are representative of the population.
- Currently in the UK there is no guidance on the management of suspected large-for-gestational age pregnancies, meaning the gestation of delivery of the standard care group is varied. Ongoing analysis of data from participants already involved shows the median gestation of delivery is 39+4 weeks' gestation.

For peer review only

INTRODUCTION

Shoulder dystocia occurs when an infant's head has been delivered vaginally and the shoulder becomes stuck behind a woman's pubic bone. This can lead to maternal and fetal complications. Maternal complications include haemorrhage, third- and fourth-degree perineal tears and psychological sequelae. Infant complications include fractures of the clavicle and humerus, brachial plexus injury, hypoxic ischaemic encephalopathy and death(1-3). Shoulder dystocia and its complications are common indications for litigation in obstetrics with settlements dealt with by the UK NHS Litigation Authority (now called NHS Resolution) from 250 cases between 2000 to 2010 costing over £100 million(4).

Fetal macrosomia is a well described risk factor for shoulder dystocia(5). This is variably defined as a neonatal birthweight >4.0Kg or 4.5Kg, or >90th customised or non-customised fetal weight centile. Preventative measures start with antenatal awareness of risk factors including fetal growth and size, maternal obesity, and diabetes.

Earlier delivery is likely to reduce the birthweight of the infant and mitigate the main risk factor for shoulder dystocia. However, it is uncertain whether this strategy would work to reduce shoulder dystocia and its associated complications, and what effect this might have on caesarean section rates and maternal complications after delivery. Research into prevention by induction is timely, in light of conflicting messages. The Royal College of Obstetricians and Gynaecologists (RCOG) does not currently recommend induction of labour for women with a suspected macrosomic fetus in the absence of diabetes(6). However, two systematic reviews and meta-analyses found that induction of labour reduced the risk of shoulder dystocia in women who had a macrosomic fetus(7, 8). Both reviews were largely based upon the 2015 randomised controlled trial by Boulvain and colleagues of 822 pregnancies with a fetus with an estimated weight greater than the 95th centile(9). While inducing labour may reduce the risk of shoulder dystocia, it has not been shown to decrease adverse neonatal sequelae and induction is associated with a marginal increased risk of operative delivery(10).

The management of large-for-gestational age (LGA) and macrosomic pregnancies in obstetrics was the focus of a landmark legal case heard by the UK Supreme Court in 2014(11).

For peer review only

Mrs Montgomery had type 1 diabetes and had a macrosomic baby, she was concerned about delivering her baby vaginally, but was not adequately informed of the risk of shoulder dystocia. During the delivery, shoulder dystocia occurred leading to a 12-minute delay in delivering the infant's body. Her son suffered from hypoxic ischaemic encephalopathy. A case was made that as Mrs Montgomery was not adequately informed of the risk of shoulder dystocia and its associated complications, and the alternative modes of delivery, namely caesarean section, she could not make a well-informed decision about the delivery of her son, therefore there was negligence in consent. After failed appeals at the Court of Session and the Inner house the case was finally heard at the UK Supreme court. The Supreme Court judgment in this case highlighted the obligation of clinicians to explain the risks and benefits of all treatment options, including that of no treatment, to women for them give a valid consent. It is therefore imperative to have robust evidence from randomised controlled trials on which to base these discussions. An investigation into the value of induction to reduce the incidence of shoulder dystocia in women with a suspected macrosomic fetus will give women and clinicians the information they need in planning their mode of delivery.

The research question is 'does induction of labour at 38^{+0} to 38^{+4} weeks' gestation, in pregnancies with suspected LGA fetuses, reduce the incidence of shoulder dystocia?'.

This manuscript describes the trial design, setting, participants and recruitment, the intervention and control groups, randomisation, outcome measures, sample size, ethical considerations, and dissemination. A separate manuscript will detail the statistical analysis plan, trial process evaluation and health economic analysis plan.

For peer review only

STUDY OBJECTIVES

Primary Objective

The primary objective is to determine the effectiveness of induction of labour at 38^{+0} to 38^{+4} weeks' gestation in reducing the incidence of shoulder dystocia in suspected LGA fetuses.

Secondary Objective

Secondary objectives are to collect comparative data on intrapartum, perinatal, infant, maternal obstetric and long-term maternal outcomes. We will collect comparative data on maternal perceptions of their labour/birth care and physical and psychological health at two and six months postnatally. We will report composite outcomes for intrapartum birth injury, prematurity associated problems and maternal intrapartum complication.

For peer review only

For peer review only - http://bmjopen.bmj.com/site/about/guidelines.xhtml

METHODS AND ANALYSIS

This protocol manuscript was written in concordance with the SPIRIT guidelines (12).

Trial Design

The Big Baby Trial is a multicentre, prospective, individually randomised controlled trial of induction of labour at 38^{+0} to 38^{+4} weeks' gestation versus standard care of fetuses that are LGA according to ultrasound scan at 35^{+0} to 38^{+0} weeks' gestation. Our definition of LGA is an estimated fetal weight >90th customised fetal weight centile using the woman's own customised Gestation Related Optimal Weight (GROW) chart(13). These charts provide the standard for assessment of fetal growth and newborn size, are recommended by RCOG Green Top Guidelines(14) and are in use in approximately 76% of NHS Trusts and Health Boards. GROW charts adjust for maternal height, weight in early pregnancy, parity, ethnic origin, and gender where known. Pathological variables such as diabetes and smoking are not adjusted for(13, 15). The GROW 90th customised centile identifies more babies at risk of adverse outcomes than LGA by conventional standards(16-19). Furthermore, GROW has been shown to be a better predictor of shoulder dystocia than the UK-WHO birthweight standard(20).

There is a parallel cohort study for women who decline randomisation but wish to participate in research. This cohort includes two sub-groups. The first is women who request a planned caesarean section. The second is women who request to be delivered by early induction of labour or expectant management. The primary objective of the cohort study is to provide comparative data on those who choose planned caesarean section and confirm generalisability of the baseline data and primary outcome with the main trial.

The trial is conducted and managed by the Warwick Clinical Trials Unit and sponsored by the University Hospitals Coventry and Warwickshire NHS Trust. Funding is provided by the National Institute for Health Research (NIHR) following a commissioned call from the Health Technology Assessment Programme (HTA study reference 16/77/02). The trial is being conducted in accordance with the principals of the Declaration of Helsinki and Good Clinical Practice (GCP).

Trial Setting

For peer review only - http://bmjopen.bmj.com/site/about/guidelines.xhtml

Although we initially planned to recruit from 60 NHS Trusts over the course of the trial to enable us to enhance recruitment, this approach has changed. We now aim to recruit 80 NHS Trusts across the UK that use customised GROW charts. Staff participating in the trial must demonstrate and document a willingness to comply with the protocol, the principles of GCP and regulatory requirements. Furthermore, they must be prepared to participate in training and adhere to the protocol.

Participants and Recruitment

Inclusion Criteria

The study participants are women aged ≥18 years with a fetus above the 90th customised GROW fetal weight centile on ultrasound scan at 35^{+0} to 38^{+0} weeks' gestation with a cephalic presentation.

Exclusion Criteria

Box 1 lists the exclusion criteria for the study.

Box 1 – Exclusion Criteria

Exclusion Criteria
Multiple pregnancy
Pregnancy with a breech or transverse lie position
Contra-indication to induction of labour
A fetus with a known serious abnormality
A home birth or elective caesarean section already planned
A caesarean section or induction indicated due to other health conditions such as cardiac disease or hypertensive disorders
Women taking medications and/or insulin therapy for diabetes or gestational diabetes (women with these conditions who are not taking medication are eligible)
A current diagnosis of a major psychiatric disorder requiring antipsychotic medication
A previous stillbirth or neonatal death ≤28days
A current intrauterine fetal death
Prisoners

For peer review only

Women unable to give informed consent e.g. learning or communication difficulties that prevent the understanding of the information provided

Recruitment

Figure 1 describes the pathway women will take through the trial and the expected number of women at each stage. Women are identified based on an ultrasound scan, performed either as part of serial fetal growth assessment or for a different indication. If the fetus has an estimated fetal weight $>90^{th}$ customised centile from 28^{+0} – 38^{+0} weeks' gestation, the woman can be approached and offered information about the study. Women are informed of the risks and benefits of participating and the possible risks and benefits of other delivery options. These can be found in the participant information sheet (supplementary material). The participant information sheet and participant consent form have been assessed for clarity by the Plain English Campaign and a Crystal Mark obtained for these. By approaching women from 28^{+0} weeks' gestation, they have time to consider their participation, ask questions to health care professionals and discuss the trial with their family and friends.

The obstetrician, or consultant midwife in charge of the woman's care is asked to provide 'obstetric confirmation', to confirm they agree for their patient to participate in the trial and receive either induction of labour or standard care. This confirmation must be completed before randomisation. To be eligible a confirmatory ultrasound scan must be performed between 35^{+0} – 38^{+0} weeks' gestation. If the fetus has an estimated fetal weight $>90^{th}$ customised GROW centile during this gestation interval and fulfils the other eligibility criteria, the woman can participate in the trial.

Intervention and Control

Intervention

Data from the West Midlands Perinatal Episode Electronic Record (PEER) database of 161,936 pregnancies found that the median length of pregnancy for LGA fetuses was 39^{+4} weeks' gestation (277 days). We further ascertained that the weekly increment of fetal weight gain in LGA pregnancies is approximately 200g. In the trial conducted by Boulvain and colleagues, the difference in fetal weight between the induction and expectant management groups was 287g(9). Based on this, we expect that for a difference of 300g between the intervention and

For peer review only

control arms, an interval of 1.5 weeks is required. Therefore, the intervention window for induction of labour is set at 38^{+0} to 38^{+4} weeks' (266-270 days) gestation. This will ensure an approximate average of eleven days separation in gestation days between groups. Induction prior to this window may decrease the risk of shoulder dystocia but would increase the risk of neonatal complications(21-23). The method of induction is by the usual practice at the participating site Trust.

Control

The control is standard care. In the UK there is no guidance on mode and timing of birth in LGA pregnancies, with practice varying from hospital to hospital and clinician to clinician. Standard care for this trial is what is provided by that hospital. The trial data monitoring and ethics committee (DMEC) continue to review the gestation of delivery of the standard care arm and so far, the median gestation of birth in the standard care arm is 39^{+4} weeks' gestation.

Outcome Measures

Primary Outcome

The primary outcome measure is the incidence of shoulder dystocia, defined by the RCOG as 'a vaginal cephalic delivery that requires additional obstetric manoeuvres to deliver the fetus after the head has delivered and gentle traction has failed'(6). These data are being extracted from clinical notes.

As the sites are unblinded, all delivery notes are reviewed by an independent expert panel to confirm if shoulder dystocia has occurred. The independent panel consists of a senior obstetrician, a senior neonatologist, a senior midwife, and a trainee obstetrician. Delivery notes are anonymised. The independent panel is blind to the trial allocation. Two panel members review each set of notes and categorise the notes into: 1. delivered by caesarean section; 2. no shoulder dystocia; 3. shoulder dystocia; or 4. needs more clarification. Where more clarification is needed, additional information is being sought from trial sites. If there is discrepancy between panel members, the entire panel discusses the case until a consensus decision is made.

For peer review only - http://bmjopen.bmj.com/site/about/guidelines.xhtml

Secondary Outcomes

The secondary outcomes are grouped into maternal peripartum, fetal peripartum, neonatal outcomes and longer-term outcomes. The secondary outcomes captured from the admission for delivery are defined in Box 2.

Box 2. Secondary Outcomes

Maternal Peripartum	Fetal Peripartum	Neonatal
Duration of hospital stay prior to delivery	Time recorded between delivery of the head and delivery of the body	Neonatal death
Duration of hospital stay after delivery	Time in labour ward	Birthweight
Mode of delivery	Time from commencement of the active second stage of labour until fetal expulsion	Gestation at birth
Perineal tears	Stillbirth	Apgar score at five minutes
Vaginal and cervical lacerations		Fractures
Primary postpartum haemorrhage		Brachial plexus injury
Clinician defined sepsis		Clinician defined sepsis
Fever >38.0°C given antibiotics		Given antibiotics
Retained placenta		Admission to the neonatal unit (intensive, special or transitional care)
Uptake of breastfeeding		Duration of hospital stay
Hospital readmission within 30 days of postnatal inpatient discharge		Hypoxic ischaemic encephalopathy
Death		Use of phototherapy
		Respiratory morbidity
		Hypoglycaemia

Randomised participants and participants in the cohort study opting for an elective caesarean section are asked to complete questionnaires at two and six months postpartum. The outcomes for the infants are assessed according to the proportion under specialist medical care at two months for a problem related to intrapartum experience, maternal report of infant health concerns at six months, in hospital healthcare costs and hospital readmission

For peer review only - http://bmjopen.bmj.com/site/about/guidelines.xhtml

within 30 days of postnatal inpatient discharge. Responses from these questionnaires identify infants who have sustained a potential birth-related injury. Relevant data related to the injury are being requested from sites and an independent adjudication committee will classify these as delivery / not delivery related. This will be undertaken by the same independent adjudication committee that is to review the delivery notes. Box 3 details the longer-term maternal and neonatal outcomes.

Box 3. Longer-term Maternal and Neonatal Outcomes

**Longer-term Outcomes**
Maternal experience (six simple questions) at two months(24)
Duration of exclusive breastfeeding at two and six months
Health-related quality of life (EQ-5D-5L) at two and six months(25)
Edinburgh Postnatal Depression Scale score at two and six months(26)
Impact of Events Scale at two months(27)
Postpartum bonding questionnaire at two months(28)
Maternal report of infant health at two and six months
Urinary incontinence ICIQ-UI short form at two and six months(29)
Faecal incontinence at two and six months
Sexual function at six months
Maternal and infant death at six months from HES-ONS linked mortality data
Participants health resource used for the economic analysis for mother and baby at two and six months

The three composite outcomes are:

1. Peripartum birth injury - includes one or both of fractures or brachial plexus injury.
2. Prematurity associated problems which include one or more of phototherapy, clinician defined sepsis before discharge from hospital, or respiratory support
3. Maternal peripartum complications which include one or more of 3rd and 4th degree perineal tears, vaginal/cervical lacerations, clinician defined sepsis before discharge from hospital or primary postpartum haemorrhage.

Sample Size

The true incidence of shoulder dystocia in women with a fetus >90th customised GROW centile is unknown. In the trial by Boulvain and colleagues on suspected macrosomia, the incidence of shoulder dystocia, defined as 'difficulty with delivery of the shoulders not resolved by McRoberts manoeuvre', in the control arm was 16/411 (3.9%)(9). In the Big Baby Trial, we

For peer review only

For peer review only - http://bmjopen.bmj.com/site/about/guidelines.xhtml

have used a similar definition of shoulder dystocia, and have estimated the incidence of shoulder dystocia in the control group to be 4%. Boulvain et al. found a relative risk for significant shoulder dystocia in the intervention group to be 0.32 (95% CI 0.12-0.85)(9). Considering this, we have set the effect size to 50% reduction in the primary outcome to 2%. This reduction is considered clinically worthwhile. To achieve a 50% reduction in the primary outcome at a 5% significance level with 90% power, 1,626 women would need to be allocated to each arm, with a sample size of 3252 women.

The sample size for this trial has been increased from 3,252 by 23% to 4,000. This is to allow for some women giving birth prior to the intervention, and to account for uncertainty in the event rate in the control group. In the trial by Boulvain and colleagues, 31/408 women (7.6%) gave birth prior to the intervention(9). The increase in the sample size also takes into account the unknown incidence of the primary outcome, an expected small loss of primary outcome, and any effect of clustering at site - although an unpublished analysis of national Growth Assessment Protocol (GAP) data by the Perinatal Institute indicated the intra-cluster correlation coefficient for being LGA to be <0.00055, suggesting that any effect will be negligible.

The trial DMEC are presented with a closed and open report of the data every six months of the study. A key event analysis was undertaken once primary outcome data were collected for 1,000 participants, given the uncertainty in the sample size estimate. The DMEC was asked to advise if a sample size adjustment was required based upon the incidence of shoulder dystocia in the control arm. These data were available on the 5th February 2020 and were considered by the DMEC who were unanimous in their satisfaction of the original planned target and recommended that the trial continues to recruit the planned 4,000 women.

Internal Pilot, Process Evaluation and Qualitative Interviews

Recruitment was assessed when ten sites had been recruiting for three months. A formative process evaluation was undertaken to assess barriers to recruitment of sites and participants and barriers to follow-up. This included interviews with ten clinicians to explore adherence to study protocol, impact on workload and impact of the trial on the woman's decision-making

For peer review only

For peer review only - http://bmjopen.bmj.com/site/about/guidelines.xhtml

process. Feedback from the pilot study and process evaluation allowed us to run seamlessly into the main study. This will be described in a further manuscript.

Randomisation

Randomisation is provided by Warwick Clinical Trials Unit using an online web application or telephone. Women are randomised using minimisation, balancing site, fetal weight centile (≤95th or >95th estimated fetal weight centile) and maternal age (≤35 or >35 years of age). To ensure allocation concealment, randomisation only takes place once all the baseline data have been collected. Women are randomised to either booking of induction of labour between 38^{+0}-38^{+4} weeks' gestation or to standard care. Women are immediately informed of the allocation.

Data Collection

Anonymised data are entered into a secured password protected trial database, developed by the programming team at Warwick Clinical Trials Unit, either at site or by the Warwick Clinical Trials Unit. Participants are identified by a unique study number. All data are stored securely and held in accordance with the relevant UK data protection legislation.

The baseline data collected are maternal height, weight, age, parity, ethnic origin, previous obstetric history, current obstetric history, tobacco use and use of antenatal corticosteroids. Women are asked to complete the EQ-5D-5L health-related quality of life questionnaire(25), Edinburgh Postnatal Depression Scale score(26), urinary incontinence ICIQ-UI short form(29), and questions on faecal incontinence and sexual function at baseline.

The fetal and neonatal outcomes collected are detailed in Box 2. In addition, we are collecting data on the proportion of infants under specialist medical care at two months for a problem related to intrapartum experience, a maternal report of infant health at six months and in-hospital costs. The maternal outcomes collected are described in Box 2. Longer-term maternal outcomes to be collected are described in Box 3.

Follow-up questionnaires are sent to participants at two- and six-months postpartum. We check the hospital electronic record for notification of a neonatal death in all infants

For peer review only - http://bmjopen.bmj.com/site/about/guidelines.xhtml

participating in the study who were discharged home, prior to sending the follow-up questionnaires. All study related data are stored in accordance with all applicable regulatory requirements and access is restricted to authorised personnel. Trial records and associated documentation will be archived for 25 years for the randomised participants and ten years for the cohort participants.

For the parallel cohort we collect the same baseline data as the randomised controlled trial. For women requesting a planned caesarean section we collect the same maternal, neonatal, and infant outcomes as the randomised controlled trial. There is a limited data collection for women in the cohort study who request induction or standard care. Women have been consented to be approached for longer-term follow up.

Data Analysis

All analyses will be by intention to treat at the time of randomisation. The primary analysis will compare the incidence of shoulder dystocia between the intervention and control groups. The comparison will be made using logistic regression models both unadjusted and adjusted for appropriate covariates. Other secondary binary outcomes will be assessed in a similar way. Continuous outcomes will be analysed using linear regression models; both adjusted and unadjusted analyses will be computed. A description of the data analyses are described in a further manuscript.

For peer review only

ETHICS AND DISSEMINATION

Ethical Conduct of the Trial

The trial complies with all UK legislation and Warwick Clinical Trials Unit standard operating procedures. Health Research Authority approval and NHS Trust R&D approval was obtained before participants were enrolled in the trial. The trial's International Standard Randomised Controlled Trial number is 18229892.

A key ethical challenge in this trial was to ensure that robust informed consent was obtained from participants. The trial requires women to consent to being randomised to a specific management pathway for the birth of their child rather than the standard clinical practice of a shared decision-making process with their clinician. It was therefore an imperative to provide the best possible information to women about the risks and benefits of all management options so they could make an informed decision about trial participation in the wider context of decision-making about their clinical care. In developing our information materials and consent processes we were guided by the standard set by the Supreme Court judgment in Montgomery(11). The key steps we took to develop the information and consent processes were:

- A review of all relevant literature from the RCOG, National Institute for Health and Care Excellence and other published works.
- Development of participants facing materials with the patient and public involvement representatives.
- A thorough peer review of all participant facing materials by obstetricians.
- The inclusion of a cohort group to respect the woman's preferred choice.

For peer review only

Adverse Event Management

Adverse events are being collected from the time of randomisation until delivery. Serious adverse events are being collected from the time of randomisation until 30 days after initial discharge following delivery. Adverse events and serious adverse events are being identified when collecting outcome data or when completing the two-month follow-up questionnaires.

For the trial only, adverse events affecting the woman or her baby which could be potentially related to the pregnancy, delivery or care of the neonate are being collected. Adverse events

are being collected for all participants in the randomised controlled trial and participants in the cohort study requesting an elective caesarean section.

Serious adverse events are only being collected for participants in the randomised controlled trial and need to be reported to Warwick Clinical Trials Unit within 24 hours of the site being made aware of the event. Certain events that would meet the definition of serious adverse events are common in pregnancy and for this trial do not need to be reported as serious adverse events. These events are being reported in the trial case report forms and comparative rates will be monitored by the DMEC. Serious adverse events that require immediate reporting for the woman and neonate are described in Box 4.

Box 4: Serious adverse events that require immediate reporting for the woman and neonate

**Maternal Serious Adverse Events**	**Neonatal Serious Adverse Events**
Maternal death	Stillbirth
Inpatient admission to intensive care and/or high dependency unit at any time during pregnancy/postnatal period	Infant death
Readmission to hospital within 30 days of initial postnatal discharge	Inpatient admission to the neonatal unit
Antenatal hospital admission not related to pregnancy	Inpatient readmission to hospital within 30 days of initial postnatal discharge*
Transfer out of the maternity unit for further inpatient care	
Inpatient admission to a mental health unit	
Symphysiotomy	

*Except for respiratory tract infection, jaundice, urinary tract infection, weight loss lasting less than 5 days, reflux and constipation.

For all serious adverse events a clinical assessment of causality is being made by a medical doctor as to whether the event is related to the booking of induction of labour. If the site or sponsor determine that there is a possible, probable, or definite relationship to the intervention then an assessment of expectedness is completed. Related and unexpected serious adverse events are expedited to the Health Research Authority Research Ethics Committee, the sponsor and the chairs of the Trial Steering Committee and DMEC.

Monitoring

For peer review only

For peer review only - http://bmjopen.bmj.com/site/about/guidelines.xhtml

All clinicians involved in obtaining consent are required to have completed GCP training. A programme of training is being delivered to all staff participating in the trial at site level. Data entered onto the trial database are being checked for accuracy and completeness by Warwick Clinical Trials Unit in accordance with the trial data management plan. A risk assessment is being undertaken and forms the basis of the trial monitoring plan. Following site initiation, the trial team is in regular contact with sites.

Patient and Public Involvement

Karen Hillyer (Chair) and Jackie Dewdney (Board Member) of the Erb's Palsy group are actively involved in the planning and development of this trial. The Erb's Palsy group is a UK-based not for profit organisation which offers advice, support and information to families affected by Erb's Palsy. Karen and Jackie led on the development of all patient-facing materials. As co-applicants they are involved in all aspects of the trial and will help inform the interpretation of the final results and dissemination of findings.

Progress so far

The trial started recruiting on 8th June 2018. As of 17th September 2021, there are 2261 randomised participants and 1566 cohort participants. Recruitment was paused on the 23rd March 2020 because of the COVID-19 pandemic. This restarted on a site-by-site basis depending on site capacity from 22nd May 2020.

Dissemination

The trial results will be reported in the NIHR journals library and published in an open access peer reviewed journal. Findings will be made available on the University of Warwick and Perinatal Institute websites. Abstracts will be submitted to major national and international conferences. Three dissemination events will be held for key stakeholders at the end of the trial. The trial will be reported in accordance with CONSORT guidelines. All publications will be submitted to the NIHR-HTA Programme for approval prior to submission for publication.

For peer review only

For peer review only - http://bmjopen.bmj.com/site/about/guidelines.xhtml

CHANGES MADE SINCE FUNDING AGREED

Since submission of the detailed project description to the NIHR-HTA some changes have been made to the protocol and agreed by the Trial Steering Committee, and DMEC. This section details the changes made and reasons for these.

Initially we predicted we would need 60 sites to reach our recruitment target. Over the course of the trial, it was evident this would need to be increased to 80 sites to enable us to improve recruitment and reach our target of 4,000 women randomised in a timely manner. In the application to the NIHR-HTA we wanted to collect outcomes on women in the cohort study who had requested an elective caesarean section. It was decided by the Trial Management Group and Trial Steering Committee that this should be extended to include outcomes on women who decline randomisation but chose either to have an early induction of labour or expectant management. The objective of this group was to provide comparative data on those who choose the timing of the birth and to confirm generalisability of the baseline data and primary outcome. Women with a current intrauterine fetal death were added to the current exclusion criteria as it is inappropriate to randomise these women and different plans would be made regarding their delivery. Prisoners were also added as a new exclusion criterion as there is a different ethical framework for their participation in medical research.

In the initial application to the NIHR-HTA we suggested that SAEs will be reported for any incidences of stillbirth, maternal death, serious intrapartum injury to the fetus or any other event that could be classified with similar severity. Once the trial had started recruiting a substantial number of SAEs were being reported that were classified as outcomes for the trial. Therefore, more formal guidance was formulated to avoid repetition in the data collection for events that did not meet the definition of SAE and to give clear instructions to the sites about what needed to be reported.

As a consequence of ongoing COVID -19 risk we are implementing a new consent process to allow for remote electronic consent rather than all consent being taken in person.

Figure 1 – Trial flow diagram with expected numbers of participants.

For peer review only

REFERENCES

1. Ahn ES, Jung MS, Lee YK, Ko SY, Shin SM, Hahn MH. Neonatal clavicular fracture: recent 10 year study. Pediatr Int. 2015;57(1):60-3.
2. Chauhan SP, Blackwell SB, Ananth CV. Neonatal brachial plexus palsy: incidence, prevalence, and temporal trends. Semin Perinatol. 2014;38(4):210-8.
3. Iffy L, Brimacombe M, Apuzzio JJ, Varadi V, Portuondo N, Nagy B. The risk of shoulder dystocia related permanent fetal injury in relation to birth weight. European Journal of Obstetrics & Gynecology and Reproductive Biology. 2008;136(1):53-60.
4. Anderson A. Ten Years of maternity claims: an analysis of the NHS Litigation Authority data - key findings. . Clinical Risk. 2013;19(1):24-31.
5. Hansen A, Chauhan SP. Shoulder dystocia: definitions and incidence. Semin Perinatol. 2014;38(4):184-8.
6. Royal College of Obstetricians and Gynaecologists. Green-top Guideline No. 42 Shoulder Dystocia. 2nd ed. London2012.
7. Boulvain M, Irion O, Dowswell T, Thornton JG. Induction of labour at or near term for suspected fetal macrosomia. Cochrane Database Syst Rev. 2016(5):CD000938.
8. Magro-Malosso ER, Saccone G, Chen M, Navathe R, Di Tommaso M, Berghella V. Induction of labour for suspected macrosomia at term in non-diabetic women: a systematic review and meta-analysis of randomized controlled trials. Bjog. 2017;124(3):414-21.
9. Boulvain M, Senat MV, Perrotin F, Winer N, Beucher G, Subtil D, et al. Induction of labour versus expectant management for large-for-date fetuses: a randomised controlled trial. Lancet. 2015;385(9987):2600-5.
10. Middleton P, Shepherd E, Crowther CA. Induction of labour for improving birth outcomes for women at or beyond term. Cochrane Database Syst Rev. 2018;5:CD004945.
11. Montgomery (Appelant) vs Lanarkshire Health Board (Respondant). Montgomery (Appelant) vs Lanarkshire Health Board (Respondant),: Supreme Court; 2015.
12. Chan AW, Tetzlaff JM, Altman DG, Laupacis A, Gotzsche PC, Krleza-Jeric K, et al. SPIRIT 2013 statement: defining standard protocol items for clinical trials. Ann Intern Med. 2013;158(3):200-7.
13. Perinatal Institute. Gestation Network Perinatal Institute2020 [Available from: https://www.gestation.net.
14. Royal College of Obstetricians and Gynaecologists. Green-top Guideline No. 31 The Investigation and Management of the Small-For-Gestational-Age Fetus. 2nd ed. London: Royal College of Obstetricians and Gynaecologists; 2013.
15. Gardosi J, Mongelli M, Wilcox M, Chang A. An adjustable fetal weight standard. Ultrasound Obstet Gynecol. 1995;6(3):168-74.
16. Larkin JC, Speer PD, Simhan HN. A customized standard of large size for gestational age to predict intrapartum morbidity. Am J Obstet Gynecol. 2011;204(6):499 e1-10.
17. Cha HH, Kim JY, Choi SJ, Oh SY, Roh CR, Kim JH. Can a customized standard for large for gestational age identify women at risk of operative delivery and shoulder dystocia? J Perinat Med. 2012;40(5):483-8.
18. Pasupathy D, McCowan LM, Poston L, Kenny LC, Dekker GA, North RA, et al. Perinatal outcomes in large infants using customised birthweight centiles and conventional measures of high birthweight. Paediatr Perinat Epidemiol. 2012;26(6):543-52.

For peer review only

For peer review only - http://bmjopen.bmj.com/site/about/guidelines.xhtml

19. Sjaarda LA, Albert PS, Mumford SL, Hinkle SN, Mendola P, Laughon SK. Customized large-for-gestational-age birthweight at term and the association with adverse perinatal outcomes. Am J Obstet Gynecol. 2014;210(1):63 e1- e11.
20. Norris T, Johnson W, Farrar D, Tuffnell D, Wright J, Cameron N. Small-for-gestational age and large-for-gestational age thresholds to predict infants at risk of adverse delivery and neonatal outcomes: are current charts adequate? An observational study from the Born in Bradford cohort. BMJ Open. 2015;5(3):e006743.
21. Engle WA. Morbidity and mortality in late preterm and early term newborns: a continuum. Clin Perinatol. 2011;38(3):493-516.
22. Dietz PM, Rizzo JH, England LJ, Callaghan WM, Vesco KK, Bruce FC, et al. Early term delivery and health care utilization in the first year of life. J Pediatr. 2012;161(2):234-9 e1.
23. Parikh LI, Reddy UM, Mannisto T, Mendola P, Sjaarda L, Hinkle S, et al. Neonatal outcomes in early term birth. Am J Obstet Gynecol. 2014;211(3):265 e1- e11.
24. Harvey S, Rach D, Stainton MC, Jarrell J, Brant R. Evaluation of satisfaction with midwifery care. Midwifery. 2002;18(4):260-7.
25. Gusi N, Olivares PR, Rajendram R. The EQ-5D Health-Related Quality of Life Questionnaire. In: Preedy V, Watson R, editors. Handbook of Disease Burdens and Quality of Life Measures. New York: Springer; 2010. p. 87-99.
26. Cox JL, Holden JM, Sagovsky R. Detection of postnatal depression. Development of the 10-item Edinburgh Postnatal Depression Scale. Br J Psychiatry. 1987;150:782-6.
27. Horowitz M, Wilner N, Alvarez W. Impact of Event Scale: a measure of subjective stress. Psychosom Med. 1979;41(3):209-18.
28. Taylor A, Atkins R, Kumar R, Adams D, Glover V. A new Mother-to-Infant Bonding Scale: links with early maternal mood. Arch Womens Ment Health. 2005;8(1):45-51.
29. Avery K, Donovan J, Peters TJ, Shaw C, Gotoh M, Abrams P. ICIQ: a brief and robust measure for evaluating the symptoms and impact of urinary incontinence. Neurourol Urodyn. 2004;23(4):322-30.

For peer review only - http://bmjopen.bmj.com/site/about/guidelines.xhtml

AUTHOR CONTRIBUTIONS

All authors read and approved the manuscript. All authors have contributed to the study design. SQ and JG are the Co-chief Investigators and oversee the running of the study. MU input into all aspects of the study design and support in running the study. LE is a Clinical Research Fellow and assisted with all aspects of the delivery of the interventions at site level. SW, KH, RG and JB managed the trial and data management. DB, EB, KF, SD, AG provided the clinician and midwifery input into the study. JF carried out the process evaluation. KB, RL and SG were the statisticians for the study. JD, KH were the Patient and Public Involvement representatives. SP and HM provided oversight of the health economic aspects of the study. A-MS was the ethicist for the study. AW and MW over sited the programming and database management and CJ was the sponsorship representative.

For peer review only

FUNDING STATEMENT

This project is funded by the National Institute for Health Research, Health Technology Assessment (NIHR HTA) project number 16/77/02. The views and opinions expressed therein are those of the authors and do not necessarily reflect those of the HTA, NIHR, NHS or the Department of Health.

For peer review only

For peer review only - http://bmjopen.bmj.com/site/about/guidelines.xhtml

COMPETING INTERESTS STATEMENT

JG is the director of the Perinatal Institute, a not for profit organisation, limited by guarantee, and a qualified provider of maternity support services to the NHS. It derives its income from some of its products and services, including the award-winning GAP program mentioned in this protocol, through which they have been able to implement training, e-learning and protocols in the majority of Trusts and Health Boards in the UK. GAP includes the standardised, RCOG endorsed customised GROW charts which will be used to identify large-for-gestational age as the entry point for this trial.

MU is chief investigator or co-investigator on multiple previous and current research grants from the UK National Institute for Health Research, Arthritis Research UK and is a co-investigator on grants funded by the Australian NHMRC. He is an NIHR Senior Investigator. He has received travel expenses for speaking at conferences from the professional organisations hosting the conferences. He is a director and shareholder of Clinvivo Ltd http://www.clinvivo.com that provides electronic data collection for health services research. He is part of an academic partnership with Serco Ltd, funded by the European Social Fund, related to return to work initiatives. He is a co-investigator on two NIHR funded studies receiving additional support from Stryker Ltd. He has accepted honoraria for teaching/lecturing from consortium for advanced research training in Africa. He was until March 2020 an editor of the NIHR journal series, and a member of the NIHR Journal Editors Group, for which he received a fee.

For peer review only

For peer review only - http://bmjopen.bmj.com/site/about/guidelines.xhtml

WORD COUNT

4251

For peer review only

For peer review only - http://bmjopen.bmj.com/site/about/guidelines.xhtml

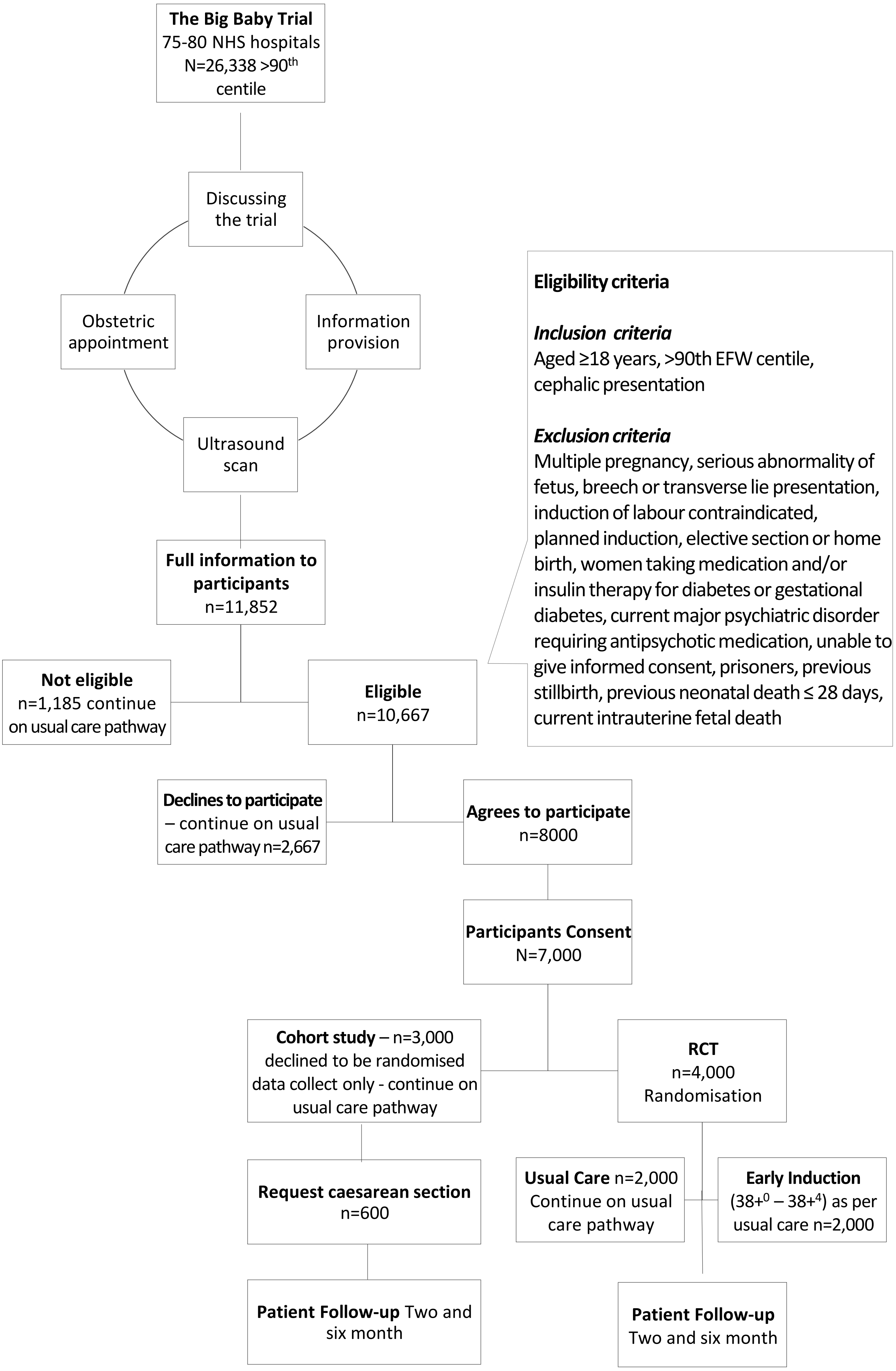
The Big Baby Trial
75-80 NHS hospitals
N=26,338 >90th centile
Discussing the trial
Obstetric appointment
Information provision
Ultrasound scan
Full information to participants
n=11,852
Not eligible
n=1,185 continue on usual care pathway
Eligible
n=10,667
Eligibility criteria
Inclusion criteria
Aged ≥18 years, >90th EFW centile, cephalic presentation
Exclusion criteria
Multiple pregnancy, serious abnormality of fetus, breech or transverse lie presentation, induction of labour contraindicated, planned induction, elective section or home birth, women taking medication and/or insulin therapy for diabetes or gestational diabetes, current major psychiatric disorder requiring antipsychotic medication, unable to give informed consent, prisoners, previous stillbirth, previous neonatal death ≤ 28 days, current intrauterine fetal death
Declines to participate – continue on usual care pathway n=2,667
Agrees to participate
n=8000
Participants Consent
N=7,000
Cohort study – n=3,000 declined to be randomised data collect only - continue on usual care pathway
RCT
n=4,000
Randomisation
Request caesarean section
n=600
Usual Care n=2,000 Continue on usual care pathway
Early Induction (38+0 – 38+4) as per usual care n=2,000
Patient Follow-up Two and six month
Patient Follow-up
Two and six month

[Print on Trust Headed Paper]

Participant Information Sheet for the Big Baby Trial

This information sheet is available in large print, audio and minority language translations. For copies, please email: BigBaby@warwick.ac.uk or download them from the website: http://warwick.ac.uk/bigbaby.

Trial title
Induction of labour for predicted macrosomia -The 'Big Baby Trial'.

'Macrosomia' refers to babies who appear to be bigger than expected.

Invitation and brief summary
Your recent ultrasound scan shows that your baby appears bigger than expected. We are inviting you to take part in a research trial to find out the best time to deliver bigger babies. We are aiming for 4000 women across the UK to take part in the research trial.

Before you decide if you want to take part in the trial, please read this information sheet carefully – it explains why the research is being done and what it means for you if you take part. One of our team will go through the information sheet with you and answer any questions you have. You can also discuss the research trial with the obstetrician or midwife looking after you.

What is the trial about?
The purpose of this trial is to find out if 'inducing' (starting) labour earlier than usual, at 38 weeks, makes it less likely that 'shoulder dystocia' will happen in women whose babies appear to be bigger than expected (over the 90th centile on the growth chart).

'Shoulder dystocia' is when the baby's head has been born but one of the shoulders becomes stuck behind the woman's pubic bone (one of the bones in the pelvis), delaying the birth of the baby's body.

'Over the 90th centile' Your growth chart is created to estimate the ideal weight your baby should be for your size and ethnicity. One in 10 babies will be bigger than expected (referred to as 'over the 90th centile').

Women who are told they may have a big baby following their antenatal ultrasound scan will not necessarily have a big baby by the time their baby is delivered.

In most cases, women with big babies have a normal labour and birth and there are no concerns. However, there is an increased chance that the birth may be more difficult, which could result in shoulder dystocia. Shoulder dystocia happens in one in 150 of all vaginal births. We know that shoulder dystocia happens more often in bigger babies, but we cannot be certain how often. We estimate that for big babies, shoulder dystocia could happen in up to one in 25 vaginal births. If shoulder dystocia happens, the midwives and doctors will use different ways to help to free the baby's shoulders, which usually allows the body to be born.

For peer review only - http://bmjopen.bmj.com/site/about/guidelines.xhtml

[Print on Trust Headed Paper]

Most babies who experience shoulder dystocia will be fine with no complications. But in around one in 10 cases of shoulder dystocia, there is stretching of the nerves in the baby's neck (brachial plexus injury). This can cause loss of movement in the baby's arm. In most cases this loss of movement is temporary, but in one in 10 of those babies the loss of movement can be permanent. In some cases, the baby may have a broken collarbone, but this heals quickly and easily in babies.

We currently do not know the best way to deliver bigger babies. It may be that starting labour earlier, when babies are smaller, means that shoulder dystocia is less likely to happen. This issue has been identified as an important unanswered question for NHS maternity units. The results of this trial will help women, midwives and obstetricians decide on the best way to deliver big babies.

What will happen if I agree to take part?

You will meet with a member of our research team at the maternity unit, either face-to-face or by telephone or video consultation, who will explain the trial. You are welcome to involve your partner, family member or friend in this discussion. We will ask you about your medical history, any previous pregnancies, and your current pregnancy. We will also review your ultrasound scan results. You can ask our team member any questions you may have. If you are eligible and would like to take part, we will ask you to either sign a consent form or to give your consent during a telephone or video call (we will then fill in the consent form for you following your verbal consent and give you a copy of this).

After you have agreed to take part in the trial and provided your consent, we will ask you to fill in questionnaires about your health, well-being and quality of life. You will be randomly selected to either have your labour induced at around 38 weeks (**the intervention group**) or to continue as normal (**the standard care group**).

If you are randomly selected to have your labour induced, your midwife or obstetrician will organise a time and date for your labour to be started (induction) and they will explain how and when this will happen.

If you are in the standard care group, you will receive the usual standard care provided by your hospital and will attend your usual antenatal appointments.

If at any point your obstetrician or midwife feels that a different plan needs to be made for your birth they will discuss this with you. If you no longer feel happy about the birth options you have as part of the trial and would like to discuss other options, you can discuss these with your obstetrician or midwife. If your birth is different to the one you were allocated, for example you have a caesarean section, you can still continue to be part of the trial.

If you agree to take part, we will collect information from your hospital records about the birth of your baby and about your and your baby's health during the time you are in hospital. We will contact you two and six months after your baby is born and ask you to fill in questionnaires about your and your baby's health and well-being. Please see page 6 of this information sheet for more details about the information we will collect.

For peer review only - http://bmjopen.bmj.com/site/about/guidelines.xhtml

[Print on Trust Headed Paper]

What are the clinical alternatives?
All women in the trial will receive the same care that they would have received if the trial was not happening. Even if you do not want to join the full trial, you can still take part in the research. (This is known as the 'cohort study'.) If you agree to join the cohort study, we would like to collect information about your baby's birth to help doctors and midwives make decisions about the best way to deliver big babies in the future. If you are happy for us to collect information about your baby's birth, we will ask you to sign a consent form.

Some women with big babies may decide that they would like their labour to be induced or that they would like to wait for it to start naturally. If you are sure that you want your labour to be induced or that you would like to wait for it to start naturally, please discuss this with your midwife and obstetrician.

If you would like to have your baby by caesarean section, you will have an opportunity to discuss this with your midwife and obstetrician. If you have a caesarean section, we would like to find out information about your birth and also about your and your baby's health after the birth. If you are happy for us to collect this information, we will ask you to sign a consent form. We will also ask you to fill in questionnaires that will include questions about your and your baby's health, well-being and quality of life. We will ask you to fill in a questionnaire when you first agree to take part in the trial and again two and six months after your baby is born. If you tell us your baby has had important health problems over this time, we will collect information about these problems from their hospital and GP records. This will help us to better understand and compare the risks and benefits of a vaginal birth or caesarean section in women with bigger babies.

What are the possible benefits of taking part in the trial?
We do not know if taking part in the research trial would benefit you or your baby. The findings will help us to advise women in the future on the best way to deliver their babies to reduce possible problems during the birth, including the risk of shoulder dystocia.

What are the possible disadvantages and risks of taking part?
Giving birth in the UK is generally very safe, whichever type of birth you have. However, if your baby is big, there can be increased risks to both you and your baby. In this research we are trying to find out the best way to reduce these risks in women who have a normal (vaginal) birth. Sometimes, obstetricians recommend a caesarean section instead of a vaginal birth, and some women may choose to have a caesarean section.

If you have a caesarean section there are different risks to consider. We have summarised what we know about the risks of vaginal births, inducing labour and caesarean sections in the tables below, but at the moment we do not know which type of birth has the fewest risks for women with bigger babies. Your midwife or obstetrician will be able to discuss the risks with you in more detail.

For peer review only - http://bmjopen.bmj.com/site/about/guidelines.xhtml

[Print on Trust Headed Paper]

Table 1 Risks of a vaginal birth with a big baby

Risks to the baby	Risks to the woman
We do not know for certain how many big babies will experience shoulder dystocia. We estimate that up to one in 25 big babies will experience shoulder dystocia and will need extra help to deliver their shoulders. Most babies who experience shoulder dystocia will have no long-term effects.	Sometimes the labour can be longer for bigger babies. In the UK, 15 in 100 women who are planning to have a vaginal birth will need to have an emergency caesarean section (please see table 3 below). Some women may need to have a forceps or ventouse (suction) delivery.
One in 10 babies who experience shoulder dystocia will have stretching of the nerves in the neck. This is called brachial plexus injury and can causes loss of movement in the baby's arm. The most common type of brachial plexus injury is Erb's palsy. For one in 10 babies with a brachial plexus injury, the loss of movement will be permanent.	Three in 100 women will have a tear to their vagina that extends into the back passage. This could affect their bowel control if the tear is not identified and repaired.
In babies who experience shoulder dystocia, one in 10 may have a fracture to their collarbone. Four in 100 babies who experience shoulder dystocia may have a fracture to their arm. These heal well.	Sometimes women with a big baby may experience heavier bleeding after the baby is born. In rare cases, some women may need a blood transfusion.
Very rarely, a baby may suffer brain damage if they did not get enough oxygen during the birth because of shoulder dystocia.	

Table 2 Risks of inducing labour with a big baby

Risks to the baby	Risks to the woman
Inducing labour at 38 weeks is safe for the baby. There is some evidence that inducing labour earlier can lead to jaundice in the baby. This usually has no long-term effects.	Often women who have labour induced will find their labour is longer and more painful than for women who go into labour naturally.
This trial aims to find out if inducing labour early, at 38 weeks, reduces the chance of shoulder dystocia. If the baby experiences shoulder dystocia, the possible complications are shown in table 1.	If you have a vaginal birth the risks are shown in table 1. Having labour induced can increase the risk of a tear to your vagina that extends into your back passage.
Babies who are born one or two weeks early are slightly more likely to need extra help at school, for example help with reading. This would affect less than 1% of babies born at 38 weeks compared with those born at 40 weeks.	Sometimes if you are being induced you may need an emergency caesarean section, and the risks of this are shown in table 3.

For peer review only - http://bmjopen.bmj.com/site/about/guidelines.xhtml

[**Print on Trust Headed Paper**]

Table 3 Risks of caesarean section

Risks to the baby	Risks to the woman
One in 10 babies may experience breathing difficulties. Some of these babies will need to have treatment for this in the neonatal unit.	Nine in 100 women report persistent pain at the wound site and in their abdomen for a few months following a caesarean section.
One to two babies in 100 will have a cut to their skin.	Five in 100 women will need to be readmitted to hospital following a caesarean section. This might be because their wound isn't healing or because they have an infection.
Some women report that it takes longer to bond with their baby after a caesarean section.	Six in 100 women will have an infection after a caesarean section. The infection may involve the scar, their bladder or kidneys, or the lining of their womb.
	One in 1000 women may have an injury to their bladder or bowel during a caesarean section. This will need repairing.
	Five in 1000 women bleed heavily (haemorrhage) during a caesarean section. Some of these women will need to have a blood transfusion. In some cases, a woman may need to have a hysterectomy (where the womb is removed) to control the bleeding.
	Five in 1000 women may need to have further surgery after their caesarean section.
	Six in 10,000 women will have a blood clot in their leg or lung following a caesarean section.
	One in four women who have a caesarean section will need another caesarean section if they attempt a vaginal birth in their next pregnancy. If you have a caesarean section and decide to try a vaginal birth in your next pregnancy, you would need extra monitoring in labour as there is a risk (one in 200 women) that the scar in the uterus can open during labour.
	If you have a caesarean section in this pregnancy, in your next pregnancy there is an increased chance of a stillbirth. This is uncommon.
	If you have a caesarean section in this pregnancy and the placenta is low in your next pregnancy, there is an increased chance that the placenta will not come away easily after the baby has been born. This can cause serious bleeding and may mean you need to have a hysterectomy. This is uncommon, but the chance increases with each caesarean section.

For peer review only - http://bmjopen.bmj.com/site/about/guidelines.xhtml

[Print on Trust Headed Paper]

What other information will you collect?
University Hospitals Coventry and Warwickshire (UHCW) are the sponsor for this trial. The trial will be managed by Warwick Clinical Trials Unit at the University of Warwick (UoW). UHCW and UoW will use information you provide and information from your hospital records and your GP records to carry out this trial. UHCW will act as the data controller for this trial, which means that they are responsible for looking after the information we collect about you and for making sure we are using it properly. UoW will act as a data processor and be under the instruction of UHCW. The trial sites are also data processors and will also be under the instruction of UHCW. You can find out more about how your information is used, how to exercise your rights relating to processing personal information, and the contact details of the data protection officer at https://www.uhcw.nhs.uk/privacy/.

We will collect information from your hospital records about the birth of your baby and about your and your baby's health during the time you are in hospital. This will include ethnic origin and health information, which is regarded as 'special category personal data'. To protect your rights, we will use the minimum amount of personally identifiable information possible. We will collect your name, date of birth, address, phone number and email address from your medical records, so we can contact you about the research and make sure that relevant information about the trial is recorded for your care, and to oversee the quality of the research. In order to do this, individuals from UHCW, UoW and regulatory authorities may review your medical notes and research records.

We will also use your information to contact you two and six months after your baby is born to ask you to fill in questionnaires about your and your baby's health and well-being, and about what, if any, healthcare services you and your baby have used. We may contact you by post, telephone, email or text message. If you tell us that your baby has had important health problems over this time, with your permission, we will collect information about these from your or your baby's hospital and GP records, or we may contact you to discuss these problems further. When we receive your questionnaire, we will review your information and if we find that any important details are missing we will contact you to collect this.

If you have given permission, we may also contact you and ask if you are willing to take part in a telephone or face-to-face interview with a member of our research team about your experiences of taking part in the Big Baby Trial. With your permission, we would also like to interview your partner or birth partner to understand what their experience of taking part in the Big Baby Trial was like.

In the UK, it is very rare that a woman dies during late pregnancy or during or after the birth (this risk is less than one in 10,000). It is also uncommon that a baby dies in the first 28 days after the birth (the risk is less than one in 400). If either of these things happens while you are taking part in this trial, it is important to us that we try to keep any distress to you and your family to a minimum. To help us achieve this, with your permission, we will check your baby's hospital records to check that your baby is alive before we invite you to fill in the two questionnaires (two and six months after the birth) or invite you to take part in an interview or further studies. When we check your baby's records, if we find that your baby has died

For peer review only - http://bmjopen.bmj.com/site/about/guidelines.xhtml

we will not contact you. It is important for us to know if you or your baby has died and if so, what caused this. To give us access to this information, we would like your permission to look at linked information held by an organisation called NHS Digital, who look after healthcare information. We would only collect this information if you did not fill in your two and six month follow-up questionnaires. We will collect your and your baby's NHS numbers from your medical records to do this.

If you have not filled in the two month follow-up questionnaire by the time that we send out the six month questionnaire, we will invite you to fill in a combined two- and six- month follow-up questionnaire six months after the birth.

If you have given us permission, we may also contact you when your child reaches age 16 to ask for their permission to keep their contact details. We would like to keep their contact details in case we want to do more research in the future.

Do I have to take part?
It is entirely up to you whether or not you take part in the trial or any other part of the research. You do not have to take part and there will be no difference in any aspects of the care that you receive if you choose not to take part. If you want to take part, you will have an opportunity to discuss this sheet with us, and ask us any questions you may have. We will then ask you to sign a consent form to confirm you have agreed to take part. Even after agreeing to take part, if you change your mind you can withdraw from the trial at any time, without having to give a reason. This will not affect the care you receive.

What happens when the research trial stops?
At the end of the trial, which will take 60 months, we will analyse the information we have collected to decide if starting labour early is the best thing to do for women and their babies. In the future, these results will help women who are expecting big babies decide if they should be induced.

Whichever part of the trial you join, we would like to keep the information we hold on you and your baby after the end of the trial. This is so that we can contact you as your baby is growing up (or we can contact your child when he or she reaches age 16) to find out if anything related to the birth has affected their longer-term health. So that we can do this, we would like your permission to look at Hospital Episode Statistics for you or your child (or both). We will collect your and your baby's NHS number to do this.

Expenses and payments
We expect that research visits will be in-line with your routine clinic appointments, so you will not need to make any extra trips to hospital. There will be no payments or travel expenses for taking part in this research.

What if I have a concern?
If you have any concerns, please talk to a member of the research team (details below) or your obstetrician or midwife. They will provide you with all the information you ask for. If you are still not happy, you should contact the<< insert as appropriate>>, who can offer

For peer review only - http://bmjopen.bmj.com/site/about/guidelines.xhtml

[Print on Trust Headed Paper]

confidential guidance on how to get independent advice. You will have the same legal rights as any other person treated in the NHS. If you or your baby is harmed by negligence you may have grounds for legal action, but you may have to pay any costs involved.

What will happen if I don't want to carry on with the trial?

Taking part in the research trial is entirely voluntary. If you do not want to continue in the trial, you can withdraw at any time without giving a reason and without it affecting your care in any way. If you decide to withdraw from the trial you can choose to have no further contact from us. However, we will keep the information about you that we have already collected if you do this. Your rights to access, change or move your information are limited as we need to manage your information in specific ways in order for the research to be reliable and accurate.

Will information about me and my baby be kept confidential?

Yes. All information we collect about you and your baby is strictly confidential. Once you have agreed to take part in the trial, we will store your contact details in a secure database which the trial team can access. Research information we collect for the trial will refer to you by a unique trial number, so the risk of you being identified is very low. We will hold contact details and research information in separate parts of the database. In rare circumstances a senior researcher from the trial team may need to pass on information we receive during the trial if there is a concern about a significant risk of harm to you or your baby, or to other people. They will only pass information to a person with authority to deal with such concerns and if possible the researcher will explain to you what information they are passing on and why. All information will be stored securely and held at the Perinatal Institute and the Warwick Clinical Trials Unit, in line with all relevant UK laws, and only authorised staff will have access to it.

When you agree to take part in a research trial, we may give information about your health and care to researchers who are running other research studies in this organisation and in other organisations. These other organisations may be universities, NHS organisations or companies involved in health and care research in this country or abroad. Organisations and researchers will only use your information to carry out research in line with the UK Policy Framework for Health and Social Care Research. The information we share will not identify you and will not be combined with other information in a way that could identify you. The information will only be used for the purpose of health and care research. It will not affect your care, and organisations and researchers cannot use it to contact you. Your information will not be used to make decisions about future services that are available to you, such as insurance.

Your rights to see, change or move your information are limited, as your information is managed in specific ways to make sure the research is reliable and accurate. If you withdraw from the trial, we will keep any information we have already collected about you. To protect your rights, we will collect as few details as possible that could identify you.

To find out more about how your information is handled, you can visit the privacy notices of the data controllers (those responsible for how and why your personal information is collected, used and held).

For peer review only - http://bmjopen.bmj.com/site/about/guidelines.xhtml

[Print on Trust Headed Paper]

www.uhcw.nhs.uk/privacy/
www.warwick.ac.uk/services/idc/dataprotection/privacynotices/researchprivacynotice

Who will be able to see my information?
Occasionally we will access your or your baby's medical records to make sure the information we have collected about you both is accurate. Only authorised staff will do this. The people who analyse the information will not be able to identify you. We will ask for your permission to tell your GP that you are taking part in the clinical trial. If you do not want us to tell your GP, you will not be able to take part.

Only authorised staff will have access to your personal details and be able to trace your identity. At the end of the study, we will store the information we collect for the trial about you and your baby for at least 25 years if you are in the randomised trial or at least 10 years for the cohort study. This is in line with UK law.

What will happen to the results of this trial?
Once the trial is complete, we will prepare and publish a report. The results will be available to the hospitals that took part in the trial. We may share information relating to the trial in scientific meetings and it may be published in scientific journals. You will not be identified in any reports or publications and none of the information will be able to be traced to you personally. The results of the trial will be published on the Big Baby website http://warwick.ac.uk/bigbaby .

Who is organising and funding this trial?
The trial is funded by the National Institute for Health Research (NIHR), Health Technology Assessment Programme. The Government set up the NIHR in 2006 to provide organised funding for research within the NHS. University Hospital Coventry and Warwickshire NHS Trust is sponsoring the trial. This covers the insurance and indemnity costs that apply to research trials. Professor Siobhan Quenby (from University Hospital Coventry & Warwickshire NHS Trust and The University of Warwick) and Professor Jason Gardosi (from the Perinatal Institute) are the chief investigators and have overall responsibility for the trial. The University of Warwick Clinical Trials Unit is organising the administration of the trial.

Who has reviewed this trial?
The trial was reviewed and approved by South West - Exeter Research Ethics Committee (REC) on 1st February 2018. The REC are an independent group of people who review all research carried out in the NHS to protect your safety, rights, well-being and dignity.

What if I want to complain?
If you want to make a complaint, please contact: Research and Development, 4th Floor Rotunda, ADA40007, University Hospitals Coventry and Warwickshire NHS Trust, Clifford Bridge Road, Coventry, CV2 2DX.

For peer review only - http://bmjopen.bmj.com/site/about/guidelines.xhtml

[Print on Trust Headed Paper]

How can I contact the hospital research team?
The hospital research team will be happy to answer any questions about the trial or your involvement in it, either now or in the future, please contact the hospital research team.
Email: <<Please insert>>
Phone: <<Please insert>>
Write to: <<Please insert>>

For more information about the 'Big Baby' trial and other useful information, please visit the Big Baby Project website: http://warwick.ac.uk/bigbaby.

Thank you for taking time to read this information sheet.

Funding acknowledgment and disclaimer - This project is funded by the National Institute for Health Research Health Technology Programme 16/77/02. The views expressed are those of the author and not necessarily those of the NIHR or the Department of Health and Social Care.

FUNDED BY NIHR | National Institute for Health Research

Crystal Mark 22637 Clarity approved by Plain English Campaign

For peer review only - http://bmjopen.bmj.com/site/about/guidelines.xhtml

SPIRIT

STANDARD PROTOCOL ITEMS: RECOMMENDATIONS FOR INTERVENTIONAL TRIALS

SPIRIT 2013 Checklist: Recommended items to address in a clinical trial protocol and related documents*

Section/item	ItemNo	Description	Page found
**Administrative information**			
Title	1	Descriptive title identifying the study design, population, interventions, and, if applicable, trial acronym	1
Trial registration	2a	Trial identifier and registry name. If not yet registered, name of intended registry	2
	2b	All items from the World Health Organization Trial Registration Data Set	2
Protocol version	3	Date and version identifier	n/a
Funding	4	Sources and types of financial, material, and other support	7,23
Roles and responsibilities	5a	Names, affiliations, and roles of protocol contributors	1
	5b	Name and contact information for the trial sponsor	7,22
	5c	Role of study sponsor and funders, if any, in study design; collection, management, analysis, and interpretation of data; writing of the report; and the decision to submit the report for publication, including whether they will have ultimate authority over any of these activities	7, 22
	5d	Composition, roles, and responsibilities of the coordinating centre, steering committee, endpoint adjudication committee, data management team, and other individuals or groups overseeing the trial, if applicable (see Item 21a for data monitoring committee)	throughout
**Introduction**			
Background and rationale	6a	Description of research question and justification for undertaking the trial, including summary of relevant studies (published and unpublished) examining benefits and harms for each intervention	4-5
	6b	Explanation for choice of comparators	10
Objectives	7	Specific objectives or hypotheses	6

For peer review only

Trial design	8	Description of trial design including type of trial (eg, parallel group, crossover, factorial, single group), allocation ratio, and framework (eg, superiority, equivalence, noninferiority, exploratory)	7

Methods: Participants, interventions, and outcomes

Study setting	9	Description of study settings (eg, community clinic, academic hospital) and list of countries where data will be collected. Reference to where list of study sites can be obtained	7
Eligibility criteria	10	Inclusion and exclusion criteria for participants. If applicable, eligibility criteria for study centres and individuals who will perform the interventions (eg, surgeons, psychotherapists)	8
Interventions	11a	Interventions for each group with sufficient detail to allow replication, including how and when they will be administered	9
	11b	Criteria for discontinuing or modifying allocated interventions for a given trial participant (eg, drug dose change in response to harms, participant request, or improving/worsening disease)	9
	11c	Strategies to improve adherence to intervention protocols, and any procedures for monitoring adherence (eg, drug tablet return, laboratory tests)	n/a
	11d	Relevant concomitant care and interventions that are permitted or prohibited during the trial	n/a
Outcomes	12	Primary, secondary, and other outcomes, including the specific measurement variable (eg, systolic blood pressure), analysis metric (eg, change from baseline, final value, time to event), method of aggregation (eg, median, proportion), and time point for each outcome. Explanation of the clinical relevance of chosen efficacy and harm outcomes is strongly recommended	10-12
Participant timeline	13	Time schedule of enrolment, interventions (including any run-ins and washouts), assessments, and visits for participants. A schematic diagram is highly recommended (see Figure)	9, figure1
Sample size	14	Estimated number of participants needed to achieve study objectives and how it was determined, including clinical and statistical assumptions supporting any sample size calculations	12
Recruitment	15	Strategies for achieving adequate participant enrolment to reach target sample size	9

Methods: Assignment of interventions (for controlled trials)

Allocation:

For peer review only - http://bmjopen.bmj.com/site/about/guidelines.xhtml

Sequence generation	16a	Method of generating the allocation sequence (eg, computer-generated random numbers), and list of any factors for stratification. To reduce predictability of a random sequence, details of any planned restriction (eg, blocking) should be provided in a separate document that is unavailable to those who enrol participants or assign interventions	14
Allocation concealment mechanism	16b	Mechanism of implementing the allocation sequence (eg, central telephone; sequentially numbered, opaque, sealed envelopes), describing any steps to conceal the sequence until interventions are assigned	14
Implementation	16c	Who will generate the allocation sequence, who will enrol participants, and who will assign participants to interventions	14
Blinding (masking)	17a	Who will be blinded after assignment to interventions (eg, trial participants, care providers, outcome assessors, data analysts), and how	14
	17b	If blinded, circumstances under which unblinding is permissible, and procedure for revealing a participant's allocated intervention during the trial	n/a
**Methods: Data collection, management, and analysis**			
Data collection methods	18a	Plans for assessment and collection of outcome, baseline, and other trial data, including any related processes to promote data quality (eg, duplicate measurements, training of assessors) and a description of study instruments (eg, questionnaires, laboratory tests) along with their reliability and validity, if known. Reference to where data collection forms can be found, if not in the protocol	14
	18b	Plans to promote participant retention and complete follow-up, including list of any outcome data to be collected for participants who discontinue or deviate from intervention protocols	14-15
Data management	19	Plans for data entry, coding, security, and storage, including any related processes to promote data quality (eg, double data entry; range checks for data values). Reference to where details of data management procedures can be found, if not in the protocol	14
Statistical methods	20a	Statistical methods for analysing primary and secondary outcomes. Reference to where other details of the statistical analysis plan can be found, if not in the protocol	15
	20b	Methods for any additional analyses (eg, subgroup and adjusted analyses)	15
	20c	Definition of analysis population relating to protocol non-adherence (eg, as randomised analysis), and any statistical methods to handle missing data (eg, multiple imputation)	N/A
**Methods: Monitoring**			

Data monitoring	21a	Composition of data monitoring committee (DMC); summary of its role and reporting structure; statement of whether it is independent from the sponsor and competing interests; and reference to where further details about its charter can be found, if not in the protocol. Alternatively, an explanation of why a DMC is not needed	17
	21b	Description of any interim analyses and stopping guidelines, including who will have access to these interim results and make the final decision to terminate the trial	18
Harms	22	Plans for collecting, assessing, reporting, and managing solicited and spontaneously reported adverse events and other unintended effects of trial interventions or trial conduct	16
Auditing	23	Frequency and procedures for auditing trial conduct, if any, and whether the process will be independent from investigators and the sponsor	17
**Ethics and dissemination**			
Research ethics approval	24	Plans for seeking research ethics committee/institutional review board (REC/IRB) approval	16
Protocol amendments	25	Plans for communicating important protocol modifications (eg, changes to eligibility criteria, outcomes, analyses) to relevant parties (eg, investigators, REC/IRBs, trial participants, trial registries, journals, regulators)	8
Consent or assent	26a	Who will obtain informed consent or assent from potential trial participants or authorised surrogates, and how (see Item 32)	9
	26b	Additional consent provisions for collection and use of participant data and biological specimens in ancillary studies, if applicable	n/a
Confidentiality	27	How personal information about potential and enrolled participants will be collected, shared, and maintained in order to protect confidentiality before, during, and after the trial	14
Declaration of interests	28	Financial and other competing interests for principal investigators for the overall trial and each study site	24
Access to data	29	Statement of who will have access to the final trial dataset, and disclosure of contractual agreements that limit such access for investigators	in main protocol
Ancillary and post-trial care	30	Provisions, if any, for ancillary and post-trial care, and for compensation to those who suffer harm from trial participation	n/a
Dissemination policy	31a	Plans for investigators and sponsor to communicate trial results to participants, healthcare professionals, the public, and other relevant groups (eg, via publication, reporting in results databases, or other data sharing arrangements), including any publication restrictions	18

For peer review only

	31b	Authorship eligibility guidelines and any intended use of professional writers	22
	31c	Plans, if any, for granting public access to the full protocol, participant-level dataset, and statistical code	18
**Appendices**			
Informed consent materials	32	Model consent form and other related documentation given to participants and authorised surrogates	Attached
Biological specimens	33	Plans for collection, laboratory evaluation, and storage of biological specimens for genetic or molecular analysis in the current trial and for future use in ancillary studies, if applicable	n/a

*It is strongly recommended that this checklist be read in conjunction with the SPIRIT 2013 Explanation & Elaboration for important clarification on the items. Amendments to the protocol should be tracked and dated. The SPIRIT checklist is copyrighted by the SPIRIT Group under the Creative Commons "Attribution-NonCommercial-NoDerivs 3.0 Unported" license.

For peer review only
